# African swine fever virus infection enhances CD14-dependent phagocytosis of porcine alveolar macrophages to promote bacterial uptake and apoptotic body-mediated viral transmission

**DOI:** 10.1128/jvi.00690-25

**Published:** 2025-06-12

**Authors:** Fengyang Shi, Zhen Xu, Peng Gao, Yajin Qu, Xinna Ge, Yongning Zhang, Jun Han, Xin Guo, Lei Zhou, Hanchun Yang

**Affiliations:** 1 National Key Laboratory of Veterinary Public Health Safety, College of Veterinary Medicine, China Agricultural University 34752, Beijing, China; 2 Key Laboratory of Animal Epidemiology of Ministry of Agriculture and Rural Affairs, College of Veterinary Medicine, China Agricultural University 34752, Beijing, China; Northwestern University Feinberg School of Medicine, Chicago, Illinois, USA

**Keywords:** African swine fever virus, macrophage, phagocytosis, bacterial uptake, CD14, apoptotic bodies

## Abstract

African swine fever virus (ASFV) is an economically devastating pathogen that can cause fatal infections in both domestic pigs and wild boar, with monocytes and macrophages as its target cells. For macrophages, phagocytosis is a fundamental and crucial process, which is usually suppressed by the virus, impeding pathogen clearance and antigen presentation. However, it was unexpectedly found that ASFV infection enhances the phagocytic ability of primary porcine alveolar macrophages (PAMs), as evaluated using an EGFP-labeled bacterial phagocytosis model. The phagocytic processes, including cell migration, bacterial adhesion, pseudopod extension, and pattern recognition receptor (PRR) expression, in ASFV-infected PAMs were systematically investigated. In addition, the upregulated PRRs were knocked down to analyze their role in enhanced phagocytosis. CD14, a receptor of LPS and phospholipid, was identified as being upregulated by ASFV, leading to enhanced bacterial uptake. Further exploration revealed that ASFV’s genomic nucleic acid in infected PAMs activates the cGAS/STING/NF-κB pathway to increase CD14 expression. Meanwhile, the free ASFV nucleic acid released from infected PAMs can also activate CD14 expression in bystander PAMs via the TLR9 pathway, facilitating ASFV transmission via apoptotic bodies (ApoBDs). Moreover, the boosted bacterial phagocytosis in the early stages of ASFV infection potentially creates a more inflamed environment with more intense cytokine production. Here, it reveals a critical mechanism by which ASFV enhances CD14-dependent bacterial uptake in PAMs via the cGAS/STING/NF-κB and TLR9 pathways, promoting viral transmission through ApoBDs and amplifying inflammatory responses to bacterial co-infections, providing vital insights into ASFV pathogenesis and host immune manipulation.

**IMPORTANCE:**

Porcine alveolar macrophages (PAMs) are the target cells of African swine fever virus (ASFV), but how ASFV impacts their phagocytic function is less known. Here, it was discovered that the nucleic acids of ASFV can enhance the expression of CD14, a receptor of LPS and phospholipid, in infected PAMs via the cGAS/STING/NF-κB pathway, or in bystander PAMs via the TLR9 pathway. Consequently, enhanced CD14 expression facilitates the uptake of bacteria and apoptotic bodies (ApoBDs), promoting the inflammatory response and ASFV cell-to-cell transmission. It provides new insights into the innate immunity response following ASFV infection and the transmission of ASFV.

## INTRODUCTION

African swine fever (ASF) is a contagious disease that can be fatal for domestic pigs and wild boars (1). It has a history of over a century and caused devastating damage to the pig industry in outbreak countries across Africa, Europe, and Asia (2, 3). The causative pathogen African swine fever virus (ASFV) is a nucleocytoplasmic large DNA virus (NCLDV) with a double-stranded DNA genome ranging from 170 to 190 kb, containing over 150 open reading frames (4). The virus particle exhibits icosahedral symmetry, with an average diameter exceeding 200 nm, forming a multi-layered structure with a double lipid membrane (5).

ASFV primarily infects monocytes and macrophages (6). As crucial “guards” of the innate immune system, macrophages are capable of maintaining homeostasis by identifying and phagocytizing particles, such as pathogens and apoptotic cells (7). Numerous studies have indicated that viruses may suppress the phagocytic function of macrophages, impeding pathogen clearance and antigen presentation, which might worsen the disease. For instance, cytomegalovirus can limit the expression of phagocytic receptors in bone marrow-derived macrophages, inhibiting the phagocytosis of *Legionella pneumophila* (8). Human rhinovirus 16 can disrupt the F-actin network in human monocyte-derived macrophages to suppress the phagocytosis of various bacteria (7). Porcine reproductive and respiratory syndrome virus (PRRSV), another virus just as ASFV that targets porcine alveolar macrophages (PAMs), is reported to inhibit the expression of pattern recognition receptors (PRRs) in PAMs, leading to suppression of phagocytosis of *Staphylococcus aureus* (9, 10). However, if ASFV infection disturbs the phagocytic ability of PAMs, and its impact is still less known.

Phagocytosis is a complex, multi-step process, and the adhesion of the particles on the plasma membrane of the macrophage in a specific or nonspecific way is the initial event. The pathogen-associated molecular patterns (PAMPs) are recognized by PRRs of macrophages. For example, Toll-like receptors can recognize lipopolysaccharide (LPS) and lipoteichoic acid (LTA) on bacterial surfaces (11). The non-transmembrane receptor CD14 binds LPS and phospholipid with high affinity (12). CD48 can bind to FimH on Gram-negative bacteria (13). CD169 can bind to sialylated glycans on the surface of bacteria and also promote the invasion of ASFV (14). In addition, receptors such as COLEC12, SDC4, and CD163 recognize specific carbohydrate sequences on the surfaces of different pathogens (15–17). After recognition, the cooperation of multiple phagocytic receptors triggers related signaling pathways, leading to pseudopod extension for phagocytosis (18).

The phagocytosis of macrophages can be modulated through various intracellular signaling pathways, with nuclear factor-κB (NF-κB) being a crucial shuttle factor between the cytoplasm and the nucleus. As a rapid response factor, NF-κB is not only recognized for promoting host inflammatory reactions, such as the rapid upregulation of inflammatory factors like TNF-α and IL-1, in response to the infection (19–21). It also controls the expression of numerous downstream phagocytosis-related genes (22). In addition, NF-κB may modulate the expression of TLRs, CD163, and other receptors, subsequently affecting phagocytic function (23, 24). Meanwhile, NF-κB signaling is activated by numerous discrete stimuli. Viral DNA, one of these stimuli, can bind to the cytosolic DNA sensor cyclic GMP-AMP synthase (cGAS) to activate the cGAS stimulator of interferon genes (STING) pathway and can also be recognized by TLR9 in endosomes. Both of these pathways may lead to the activation of NF-κB (25, 26). However, it remains to be clarified whether the cascade effects of these pathways can regulate phagocytic function.

Early studies have illustrated that the phagocytic ability of macrophages could be affected by viral infections, which further disrupts immune functions and leads to severe consequences. As well, it was also found that PAMs can phagocytize apoptotic bodies (ApoBDs) from ASFV-infected cells to facilitate ASFV transmission (27). It will be interesting to explore whether ASFV infection changes the phagocytic ability of PAMs and the impact of these changes on PAMs’ function and viral transmission.

In this study, a phagocytosis model was initially established using PAMs and EGFP-labeled *E. coli* to analyze the influence of ASFV infection on the phagocytic ability of PAMs. The phagocytosis-related processes, genes, and regulation pathways were systematically analyzed to further explore the underlying mechanism. It was unexpectedly found that ASFV infection enhances CD14-dependent phagocytosis in PAMs, which is achieved via the cGAS/STING/NF-κB pathway in infected PAMs and the TLR9 pathway in bystander cells. This enhancement promotes viral transmission via ApoBDs and exacerbates inflammatory responses during bacterial co-exposure, highlighting critical mechanisms linking viral manipulation of host immunity to disease progression and secondary infection risks.

## RESULTS

### ASFV infection enhances the phagocytosis of PAMs

**Fig 1:**
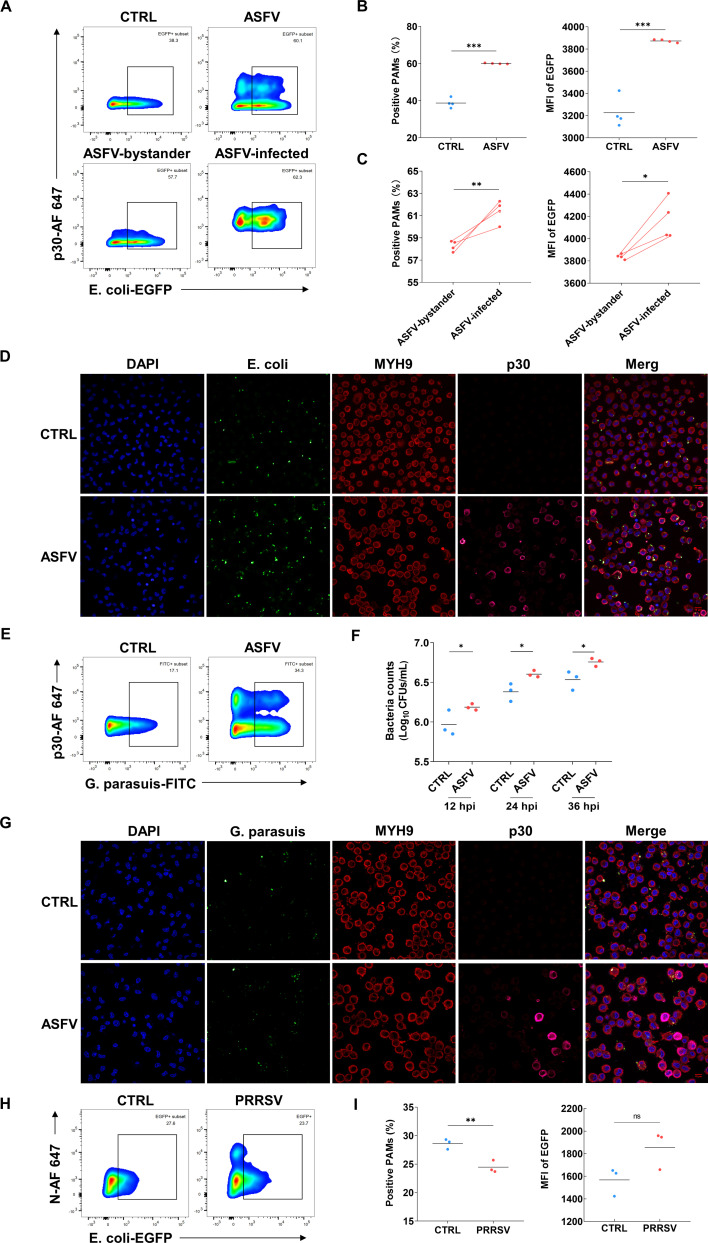
ASFV infection enhances the phagocytosis of PAMs. (**A**) Flow cytometry scatter plots of PAMs phagocytizing *E. coli*-EGFP (multiplicity of infection [MOI] 10) at 24 h after ASFV infection (MOI 0.5). Representative data from multiple experiments are shown. (**B**) The percentage and MFI of PAMs phagocytizing *E. coli*-EGFP (MOI 10) at 24 h after ASFV infection (MOI 0.5) (mean of four independent experiments ± SD). (**C**) The percentage and MFI of infected and bystander PAMs phagocytizing *E. coli*-EGFP (MOI 10) at 24 h after ASFV infection (MOI 0.5). *n* = 4. (**D**) IFA analysis of PAMs phagocytizing *E. coli*-EGFP (MOI 10) at 24 h after ASFV infection (MOI 0.5). Scale bar, 10 µm. (**E**) Flow cytometry scatter plots of PAMs phagocytizing *G. parasuis* (MOI 25) at 24 h after ASFV infection (MOI 0.5). Representative data from multiple experiments are shown. (**F**) Plate count results of PAMs phagocytizing *G. parasuis* (MOI 25) at 24 h after ASFV infection (MOI 0.5) (mean of three independent experiments ± SD). (**G**) IFA analysis of PAMs phagocytizing *G. parasuis* (MOI 25) at 24 h after ASFV infection (MOI 0.5). Scale bar, 50 µm. (**H**) Flow cytometry scatter plots of PAMs phagocytizing *E. coli*-EGFP (MOI 10) at 24 h after PRRSV infection (MOI 0.5). Representative data from multiple experiments are shown. (**I**) The percentage and MFI of PAMs phagocytizing *E. coli*-EGFP (MOI 10) at 24 h after PRRSV infection (MOI 0.5) (mean of three independent experiments ± SD).

A phagocytic model based on EGFP-labeled *E. coli* was first constructed by transforming EGFP-expressing plasmid pET-28a-EGFP, and the fetal bovine serum (FBS) concentrations, inoculation ratio of bacteria/macrophage, and phagocytic time were optimized, respectively (Fig. S1). Some previous studies have found that viruses may impair the phagocytic ability of macrophages (7, 8, 28). To assess the influence of ASFV infection on the phagocytic ability of PAMs, the established model of *E. coli*-EGFP above was used. The phagocytic rate of PAMs and the mean fluorescence intensity (MFI) of PAMs that phagocytized bacteria in the inoculation group were both significantly upregulated at 24 hpi (hours post inoculation) (Fig. 1A, B and D), as well as 12 hpi (Fig. S2A and B). And the ASFV-infected PAMs exhibited a heightened capability to phagocytize *E. coli*-EGFP compared to bystander PAMs (Fig. 1A and C), especially at 12 hpi (Fig. S2A and C). To further confirm whether this increase is the general response to the bacteria causing disease on pigs, the same experiment was carried out using *Glaesserella parasuis* (*G. parasuis*), the causative agent of Glässer’s disease, characterized by fibrinous polyserositis, arthritis, and meningitis, especially after the immune-suppressed viral infection (29). Consistent with the expectations, ASFV infection enhanced the phagocytic ability of PAMs for *G. parasuis* (Fig. 1E and G) as well. The result of plate colony counting also demonstrated that the phagocytic ability of PAMs for *G. parasuis* was significantly enhanced at 12, 24, and 36 hpi (Fig. 1F; Fig. S2D). To verify the reliability of this model, PRRSV, which has been reported to inhibit the phagocytic ability of PAMs (30, 31), was set as a control. It was found that the ability of PAMs to phagocytize *E. coli*-EGFP was significantly inhibited by PRRSV at 24 hpi (Fig. 1H and I). Meanwhile, pseudorabies virus (PRV), a large dsDNA virus, was also used as a control to verify the specificity of the results, and it was found that PRV infection had no obvious effect on the bacterial phagocytosis rate of PAMs (Fig. S2E and F) (32).

### ASFV infection does not affect the migration, bacterial adhesion, and pseudopod extension abilities of PAMs

**Fig 2:**
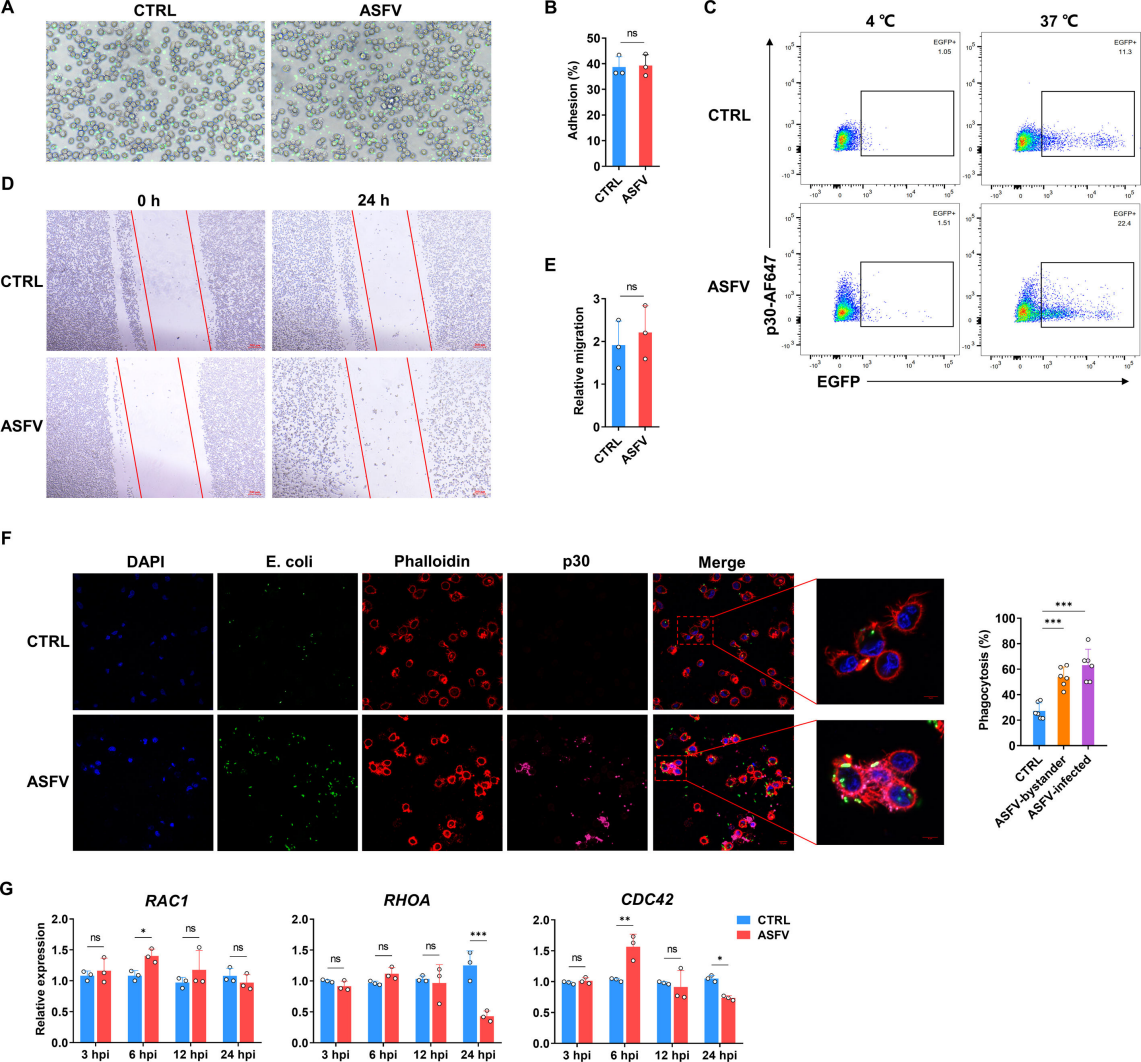
ASFV infection does not affect the migration, bacterial adhesion, and pseudopod extension abilities of PAMs. (**A**) PAMs were infected with ASFV for 24 h (MOI 0.5), then infected with *E. coli*-EGFP (MOI 10) at 4°C for 2 h. The adhesion of PAMs to *E. coli*-EGFP was observed under a fluorescence microscope. Representative data from multiple experiments are shown. Scale bar, 50 µm. (**B**) Calculate the adhesion percentage of PAMs to *E. coli*-EGFP (mean of three independent experiments ± SD). (**C**) Flow cytometry scatter plots of PAMs phagocytizing E. coli-EGFP (MOI 10) at 4°C or 37°C after ASFV infection for 24 h (MOI 0.5). The ASFV-infected PAMs and bystander PAMs are distinguished by detecting the expression of ASFV p30. (**D**) The cell scratch assay was performed on PAMs at 0 and 24 h after ASFV infection (MOI 0.5). The number of cells within the red line in the CTRL group was used as the baseline. Representative data from multiple experiments are shown. Scale bar, 200 µm. (**E**) Count the number of PAMs within the red line. The results are presented as the relative values, with 24 h compared to 0 h (mean of three independent experiments ± SD). (**F**) PAMs were infected with ASFV for 24 h (MOI 0.5), and then infected with E. coli-EGFP for 2 h (MOI 10), after which the pseudopod extension was analyzed by IFA. F-actin was labeled with Alexa 594-conjugated Phalloidin. ASFV-infected PAMs were labeled with an antibody against p30 followed by an Alexa 647-conjugated secondary antibody. Representative data from multiple experiments are shown. Calculate the percentage of PAMs that have phagocytized bacteria (mean ± SD of six samples). Scale bar, 10 µm. (**G**) The relative mRNA levels of *RAC1*, *RHOA*, and *CDC42* after ASFV infection were analyzed using qPCR (MOI 0.5). The results are expressed as the mean ± SD from three independent experiments.

Phagocytosis is a biological process that involves multiple coordinated steps (33). To explore the mechanism of ASFV activating phagocytosis in PAMs, the main process of bacterial phagocytosis was analyzed, respectively. The bacterial adhesion ability of PAMs was initially assessed at 4℃, and no obvious difference was found after ASFV infection (Fig. 2A and B). The flow cytometry results confirm that the *E. coli*-EGFP only attaches to the PAMs at 4℃ with limited internalization, but when culture temperature increases to 37℃, the ratio of EGFP^+^PAMs in the ASFV-infected group was much higher than in the control group, which was consistent with the phenomenon described above (Fig. 2C). Besides, Sibofimloc, an inhibitor targeting the bacterial adhesin FimH, did not inhibit the enhancement of phagocytosis ability of ASFV-infected PAMs (Fig. S3A and B). Considering the migration ability of PAMs might alter the efficiency of bacteria uptake, a scratch assay was also carried out in the 24-well plates, and it was found that few PAMs migrated into the scratch area, and there was no significant difference between the ASFV infection group and the mock group (Fig. 2D and E), suggesting that PAMs may be more inclined to move within a small area around them, which might not be sufficient to enhance the phagocytic ability. To further investigate whether the pseudopod extension ability changed in ASFV-infected PAMs, the pseudopod extension was directly observed by labeling the F-actin with phalloidin. Several articles have reported that ASFV leads to cell membrane damage and pseudopod rupture at the late stage of infection (27, 34), so the appropriate multiplicity of infection (MOI) and inoculation time were selected to ensure that the complete pseudopodia were observed. It was found that ASFV-infected PAMs seemed to show similar pseudopod extension as the control (Fig. S3C). And both the control and ASFV-infected groups showed active pseudopod extension in response to *E. coli*-EGFP stimulation, even though the intracellular bacterial load in the ASFV-infected group was significantly higher than that in the control group (Fig. 2F). Since pseudopod extension is the result of a cascade of activations involving multiple proteins (18), mRNA levels of key genes (*RAC1*, *RHOA,* and *CDC42*) for pseudopod extension were also tested by qPCR. And it did not show consistent significant changes (Fig. 2G). These results indicate that the enhanced phagocytic ability of ASFV-infected PAMs is not related to the pseudopod extension process.

### ASFV infection enhances the phagocytosis of PAMs by upregulating the expression of CD14

**Fig 3:**
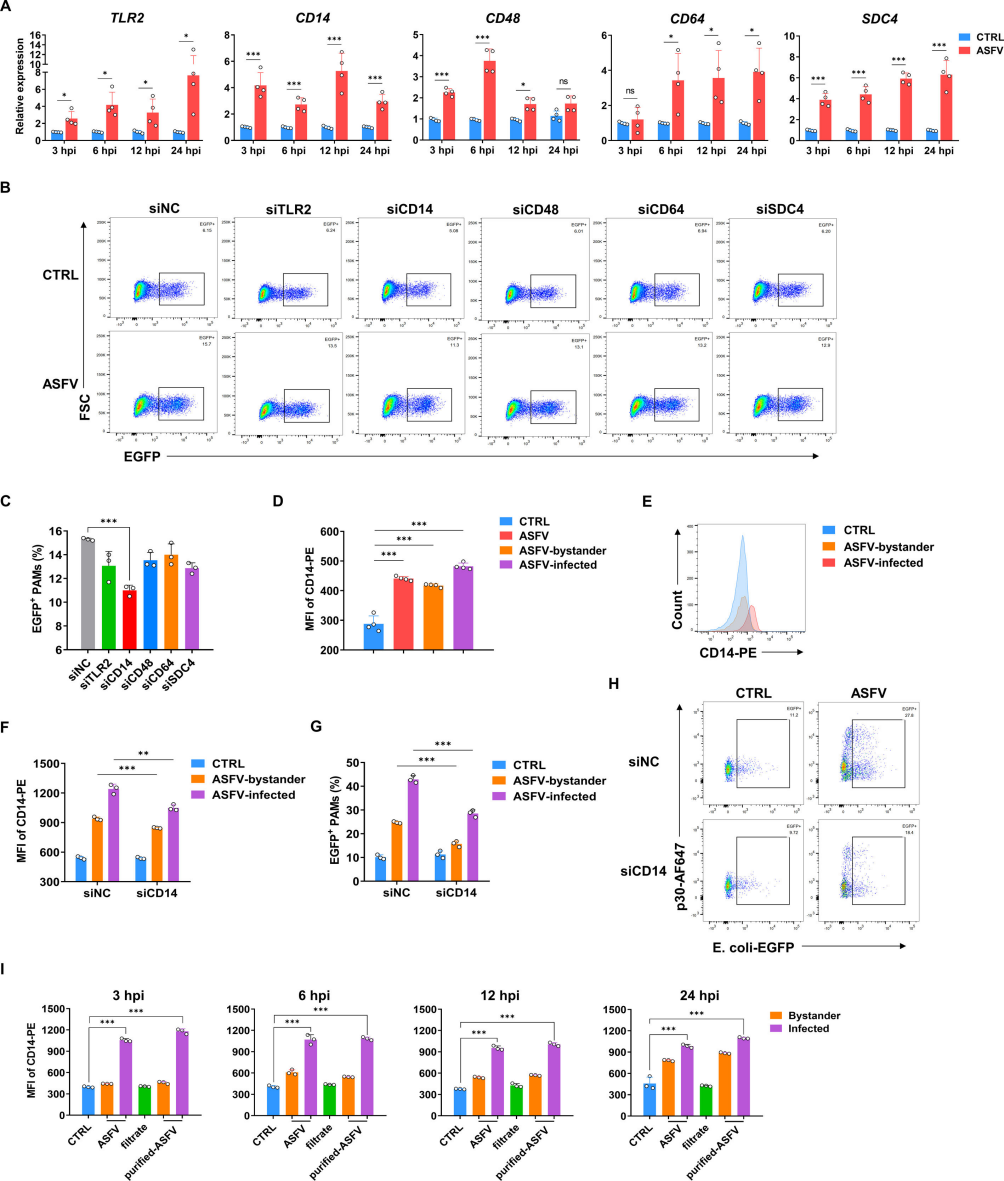
ASFV infection enhances the phagocytosis of PAMs by upregulating the expression of CD14. (**A**) The relative mRNA levels of *TLR2*, *CD14*, *CD48*, *CD64*, and *SDC4* after ASFV infection were analyzed using qPCR (MOI 0.5) (mean of four independent experiments ± SD). (**B**) PAMs were treated with different siRNAs for 24 h, then infected with ASFV for another 24 h (MOI 0.5), and subsequently infected with *E. coli*-EGFP (MOI 5) for 2 h. Flow cytometry was used to detect the phagocytosis of *E. coli*-EGFP. Representative data from multiple experiments are shown. (**C**) The phagocytic rate of PAMs on *E. coli*-EGFP (mean of three independent experiments ± SD). (**D**) MFI of CD14 expression in PAMs at 24 hpi (mean of three independent experiments ± SD). (**E**) The histogram of CD14 expression in PAMs at 24 hpi. (**F**) MFI of CD14 expression in PAMs treated with si-CD14 and then infected with ASFV for 24 h (mean of three independent experiments ± SD). The ASFV-infected PAMs and bystander PAMs are distinguished by detecting the expression of ASFV p30. (**G**) The phagocytic rate of *E. coli*-EGFP (MOI 10) of PAMs treated with si-CD14 and then infected with ASFV for 24 h (mean of three independent experiments ± SD). The ASFV-infected PAMs and bystander PAMs are distinguished by detecting the expression of ASFV p30. (**H**) Flow cytometry scatter plots of PAMs treated with si-CD14 and then infected with ASFV for 24 h. Representative data from multiple experiments are shown. (**I**) MFI of CD14 expression in PAMs infected with different ASFV viral fluid components. The group cultured with the unfiltered virus fluid is referred to as the “ASFV group.” The group cultured with the filtrate is referred to as the “filtrate group.” The group cultured with the virus that remained after filtration is referred to as the “filtered ASFV group” (mean of three independent experiments ± SD).

To further identify the factors that potentially regulate phagocytosis of PAMs post-ASFV infection, the expression level of phagocytosis-related genes was analyzed, based on the published data of single-cell RNA sequencing (scRNA-seq) from ASFV-infected PAMs and mock PAMs (35). The phagocytosis-related genes (*TLR2*, *CD14*, *CD48*, *CD64,* and *SDC4*), showing upregulated expression in the scRNA-seq data, were confirmed by qPCR to be significantly higher expressed in ASFV-infected PAMs (Fig. 3A). And they were screened out for functional confirmation. In addition, the other four important phagocytosis-related genes (*TLR4*, *CD163*, *COLEC12*, and *CD169*) did not show significant upregulation in the scRNA-seq data nor did they exhibit significant changes or consistent trends at different time points in our current test (Fig. S3D). They were not considered for further study due to their inconsistencies at different time points and conflicting with the observed phagocytic phenotype above.

To further identify the phagocytosis-related key molecules and pathways, PAMs were transfected with siRNAs targeting *TLR2*, *CD14*, *CD48*, *CD64,* or *SDC4* for 24 h, respectively, followed by infection with ASFV for another 24 h, and then their phagocytosis functions were evaluated by adding a low dose of *E. coli*-EGFP (MOI 5). The knockdown of each target did not affect the viability and ASFV infectivity of PAMs (Fig. S4A and B) and seemed to have variable effects on the phagocytic rate, but only the knockdown of CD14 resulted in a significantly decreased phagocytic rate (Fig. 3B and C). As an important receptor on the surface of the cell membrane for recognizing bacterial structures such as LPS and LTA, CD14 plays a vital role in the process of macrophage phagocytosis (36–38). So, the expression of CD14 (MFI) and the ratio of CD14^high^ PAMs, post-ASFV infection at 24 hpi, were tested by flow cytometry. The results showed that the expression of CD14 was significantly upregulated in both ASFV-infected PAMs and bystanders (Fig. 3D and E), while PRRSV or PRV infection had no such effect (Fig. S4C and D). This result has been further verified by infecting PAMs with *E. coli*-EGFP at an MOI of 10. The knockdown of CD14 was accompanied by a significant reduction in phagocytosis ability after ASFV infection (Fig. 3F through H).

Considering that the ASFV used for inoculation was cultured and harvested from PAMs, it may contain inflammatory factors that could potentially activate the phagocytosis of PAMs. To exclude the potential effects, the cultured virus was centrifuged at 10,000 *× g* for 5 min to remove cellular debris, and subsequently ultrafiltered by using a 100 kDa molecular weight cutoff filter (Millipore, Tullagreen, Ireland) to separate the major inflammatory factors. The remaining ASFV and ASFV-free filtrate were separately used to repeat the inoculation under identical conditions as described above (Fig. S4E). The results showed that treatment with ASFV-free filtrate did not enhance the expression of CD14, indicating that the upregulation of CD14 was not related to the inflammatory factors that may be present during viral incubation (Fig. 3I).

### The enhancement of CD14-dependent phagocytosis by ASFV is dependent on NF-κB activation

**Fig 4:**
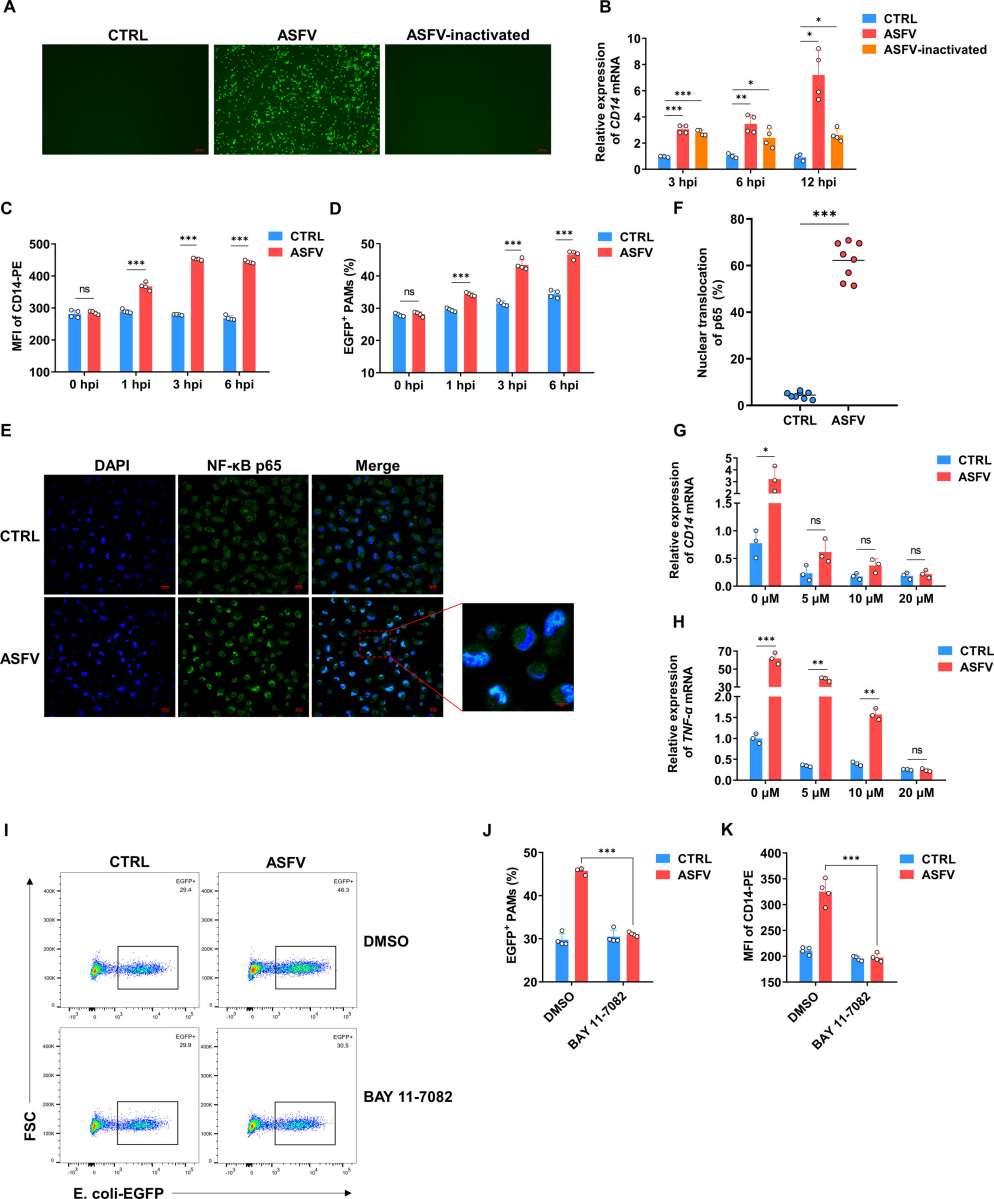
The enhancement of CD14-dependent phagocytosis by ASFV is dependent on NF-κB activation. (**A**) PAMs were infected with heat-inactivated ASFV for 24 h (MOI 1), and the infection of ASFV was observed using a fluorescence microscope. ASFV-infected PAMs were labeled with an antibody against p30, followed by an Alexa 488-conjugated secondary antibody. Representative data from multiple experiments are shown. Scale bar, 100 µm. (**B**) PAMs were infected with heat-inactivated ASFV for 3, 6, and 12 h (MOI 1). The relative level of *CD14* mRNA was analyzed by qPCR (mean of four independent experiments ± SD). (**C, D**) CD14 MFI (**C**) and the phagocytosis (**D**) in the early stage of ASFV infection in PAMs (MOI 1) (mean of four independent experiments ± SD). (**E**) IFA analysis of the nuclear translocation of NF-κB p65 in PAMs infected with ASFV for 3 h (MOI 1). NF-κB cells were labeled with an antibody against p65, followed by an Alexa 488-conjugated secondary antibody. Nuclei were stained with DAPI. Representative data from multiple experiments are shown. Scale bar, 10 µm. (**F**) Calculate the percentage of NF-κB p65 nuclear translocation in PAMs infected with ASFV for 3 h (MOI 1) (mean ± SD of eight samples). (**G, H**) PAMs were pre-treated with different concentrations of NF-κB inhibitor BAY 11-7082 for 1 h, and then infected with ASFV for 3 h (MOI 1). The relative levels of *CD14* (**G**) and *TNF-α* (**H**) mRNA in PAMs were analyzed by qPCR (mean of three independent experiments ± SD). (**I**) Flow cytometry scatter plots of PAMs phagocytizing *E. coli*-EGFP (MOI 10) after pre-treatment with BAY 11-7082 (5 µM) and subsequent ASFV infection for 3 h (MOI 1). Representative data from multiple experiments are shown. (**J, K**) The phagocytosis of *E. coli*-EGFP (MOI 10) (**J**) and CD14 expression (**K**) in PAMs that were pre-treated with BAY 11-7082 (5 µM) and subsequently infected with ASFV for 3 h (MOI 1) (mean of four independent experiments ± SD).

It is wondered how ASFV enhances the expression of CD14 and the phagocytosis of PAMs. CD14 on the membrane of infected PAMs was significantly increased as early as 3 hpi, and it did not continue to rise with prolonged infection time (Fig. 3I). Given the rapid upregulation of CD14, it is possible that this occurs before viral replication. To study whether the process of viral entry into PAMs can indeed activate CD14 transcription, we subjected the virus to heat inactivation (70°C for 30 min). As expected, heat-inactivated ASFV, which had lost infectivity (Fig. 4A), still induced an increase in CD14 transcription in the early stages of infection (Fig. 4B). The ASFV without inactivation could induce much higher CD14 transcription at 12 hpi (Fig. 4B). To further identify the active time of CD14 transcription, several earlier time points were set, and a higher MOI was used. To our surprise, significant upregulation of CD14 was detected as early as 1 hpi, reaching the peak at 3 hpi (Fig. 4C). The qPCR results indicate that it is the internalization of the virus, rather than adsorption, that stimulates the transcription of CD14 (Fig. S5A). Moreover, consistent with this trend, the phagocytosis ability of PAMs infected with ASFV was also enhanced simultaneously (Fig. 4D). As a rapid response factor in the cellular defense signal pathway, NF-κB was also reported to regulate the expression of phagocytosis-related genes (39, 40). To analyze whether it is involved in the upregulation of CD14, the distribution of NF-κB p65 after ASFV infection was first investigated. The obvious nuclear translocation of NF-κB p65 can be observed at 3 hpi after ASFV infection, indicating that NF-κB was markedly activated (Fig. 4E and F). Then, BAY 11-7082, an inhibitor of IκBα phosphorylation, was used to suppress the NF-κB activation, and the ASFV-induced CD14 transcription can be significantly suppressed, at a safe concentration without evident cytotoxicity (Fig. 4G; Fig. S5B). In this test, the TNF-α mRNA levels were also set to serve as a positive control to measure the inhibitor’s effectiveness (Fig. 4H). Since 10 or 20 µM BAY would inhibit the internalization of ASFV (Fig. S5C). To exclude the possibility of false positives due to reduced internalization of ASFV particles into PAMs, thereby leading to reduced CD14 transcription, 5 µM BAY was selected for subsequent experiments. It was found that BAY treatment completely blocked the enhancement of phagocytic ability induced by ASFV infection (Fig. 4I and J). The expression of CD14 in PAMs was reduced to the control level, regardless of whether they were infected with ASFV or not (Fig. 4K), which was consistent with the results of the inhibition of phagocytic ability. Together, these results indicate that ASFV enhances the CD14 expression and phagocytosis ability of PAMs in an NF-κB activation-dependent manner.

### ASFV enhances CD14-dependent phagocytosis through cGAS/STING/NF-κB pathways

**Fig 5:**
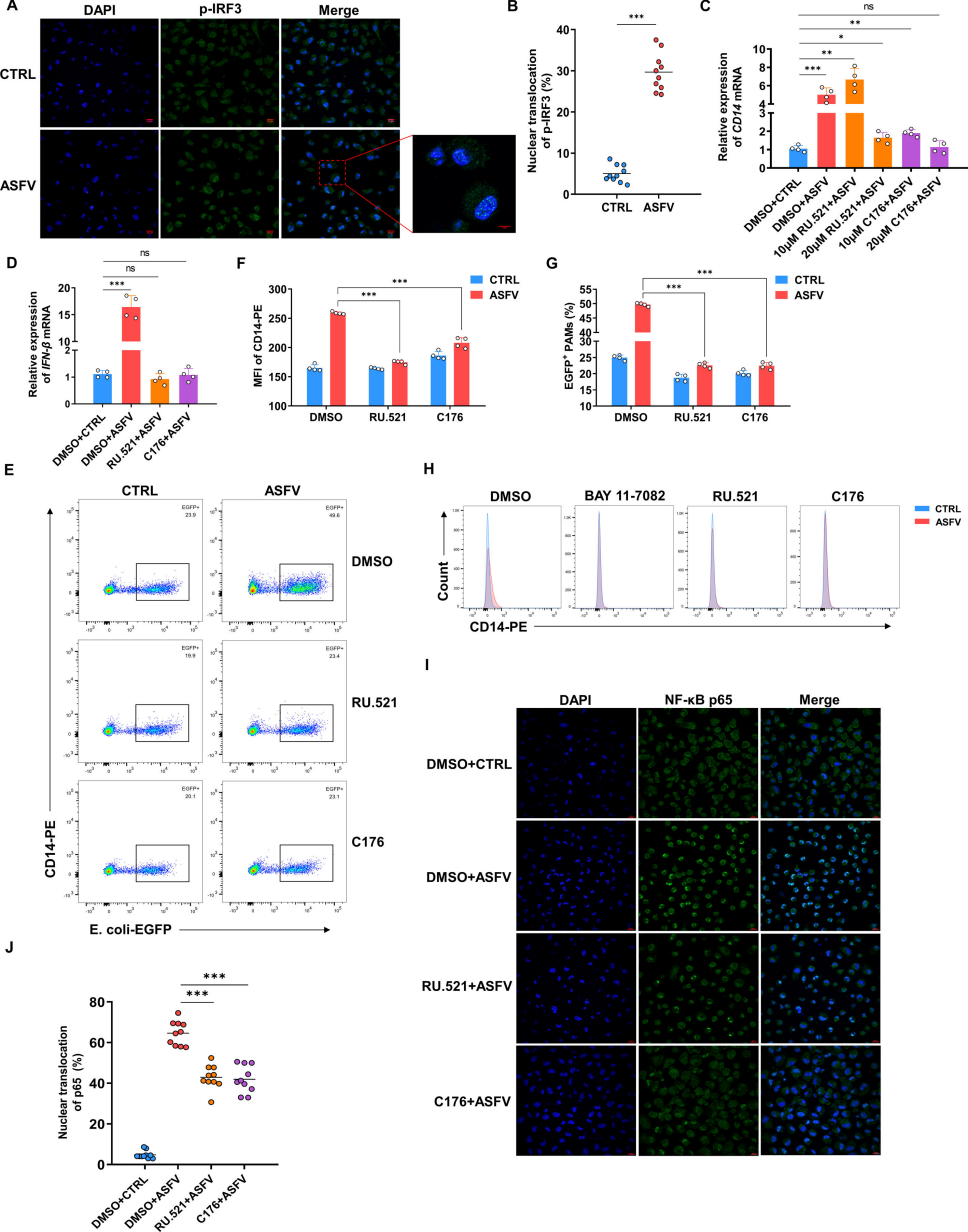
ASFV enhances the CD14-dependent phagocytosis through cGAS/STING/NF-κB pathways. (**A**) IFA analysis of the nuclear translocation of p-IRF3 in PAMs infected with ASFV for 3 h (MOI 1). p-IRF3 was labeled with its specific antibody followed by an Alexa 488-conjugated secondary antibody. Nuclei were stained with DAPI. Representative data from multiple experiments are shown. Scale bar, 10 µm. (**B**) Calculate the percentage of p-IRF3 nuclear translocation in PAMs infected with ASFV for 3 h (MOI 1) (mean ± SD of ten samples). (**C,D**) PAMs were pre-treated with different concentrations of the cGAS inhibitor RU.521 or the STING inhibitor C176 for 1 h, and then infected with ASFV for 3 h (MOI 1). The relative levels of *CD14* (**C**) and *IFN-β* (**D**) mRNA in PAMs were analyzed by qPCR (mean of four independent experiments ± SD). (**E**) Flow cytometry scatter plots of PAMs phagocytizing *E. coli*-EGFP (MOI 10) after pre-treatment with RU.521 (20 μM) or C176 (20 µM) and subsequent ASFV infection for 3 h (MOI 1). (**F, G**) CD14 MFI (**F**) and the phagocytosis of *E. coli*-EGFP (MOI 10) (**G**) in PAMs pre-treated with RU.521 (20 μM) or C176 (20 µM) and subsequently infected with ASFV for 3 h (MOI 1) (mean of four independent experiments ± SD). (**H**) The histogram of CD14 expression in PAMs after different inhibitors pre-treatment for 1 h and subsequent ASFV infection for 3 h. (**I**) IFA analysis of the nuclear translocation of NF-κB p65 in PAMs pre-treated with different inhibitors for 1 h and subsequently infected with ASFV for 3 h (MOI 1). NF-κB cells were labeled with an antibody against p65 followed by an Alexa 488-conjugated secondary antibody. Nuclei were stained with DAPI. Representative data from multiple experiments are shown. Scale bar, 10 µm. (**J**) Calculate the percentage of NF-κB p65 nuclear translocation in PAMs pre-treated with different inhibitors for 1 h and subsequently infected with ASFV for 3 h (MOI 1) (mean ± SD of 10 samples).

Considering that the upregulation of CD14 could be detected as early as 1 hpi (Fig. 4C), it should be an early event before the viral replication. Viral infection can trigger the cytoplasmic DNA sensor cGAS, recognizing cytosolic double-stranded DNA and producing the second messenger cGAMP to activate STING, possibly leading to the activation of NF-κB (41). To further analyze whether the internalized viral nucleic acids trigger this activation, the nuclear translocation of the key downstream molecule p-IRF3 in the cGAS-STING pathway was observed. It shows a significant increase in nuclear translocation after ASFV infection, suggesting the activation of the pathway (Fig. 5A and B). Then PAMs were pretreated with the inhibitors of cGAS or STING (RU.521 or C176) before ASFV infection, and it was found that the transcription level of CD14 was significantly suppressed after treatment with high concentrations of these inhibitors, with no evident cytotoxicity (Fig. 5C; Fig. S6A). Consistent with the experiment of BAY 11-7082, the ASFV internalization was also tested under the same concentrations of inhibitors to exclude the possibility that these inhibitors affect viral internalization (Fig. S6B). The mRNA of *IFN-β* was used as a positive control to measure the effectiveness of the two inhibitors (Fig. 5D). Further phagocytosis experiments revealed that both inhibitors significantly prevented the increase in CD14 on the cell membrane and the enhancement of phagocytic ability induced by ASFV (Fig. 5E through H). To prove that the results were specific to ASFV infection, we used PRV, which also activates the cGAS-STING pathway in the early stages of infection (42), to infect PAMs for 3 h, finding that the phagocytic ability did not change (Fig. S6C and D). Finally, to demonstrate that activation of the cGAS-STING pathway induced the translocation of the downstream NF-κB p65, we pretreated PAMs with RU.521 or C176 separately, then infected with ASFV and detected the nuclear translocation of p65. It was observed that both inhibitors had some inhibitory effect on the nuclear translocation of p65; however, there may be other mechanisms for the early induction of NF-κB activation by ASFV infection (Fig. 5I and J). These data indicate that ASFV can enhance CD14-dependent phagocytosis through the cGAS/STING/NF-κB pathway.

### Free viral DNA induces the upregulation of CD14 in bystander PAMs via TLR9 signaling

**Fig 6:**
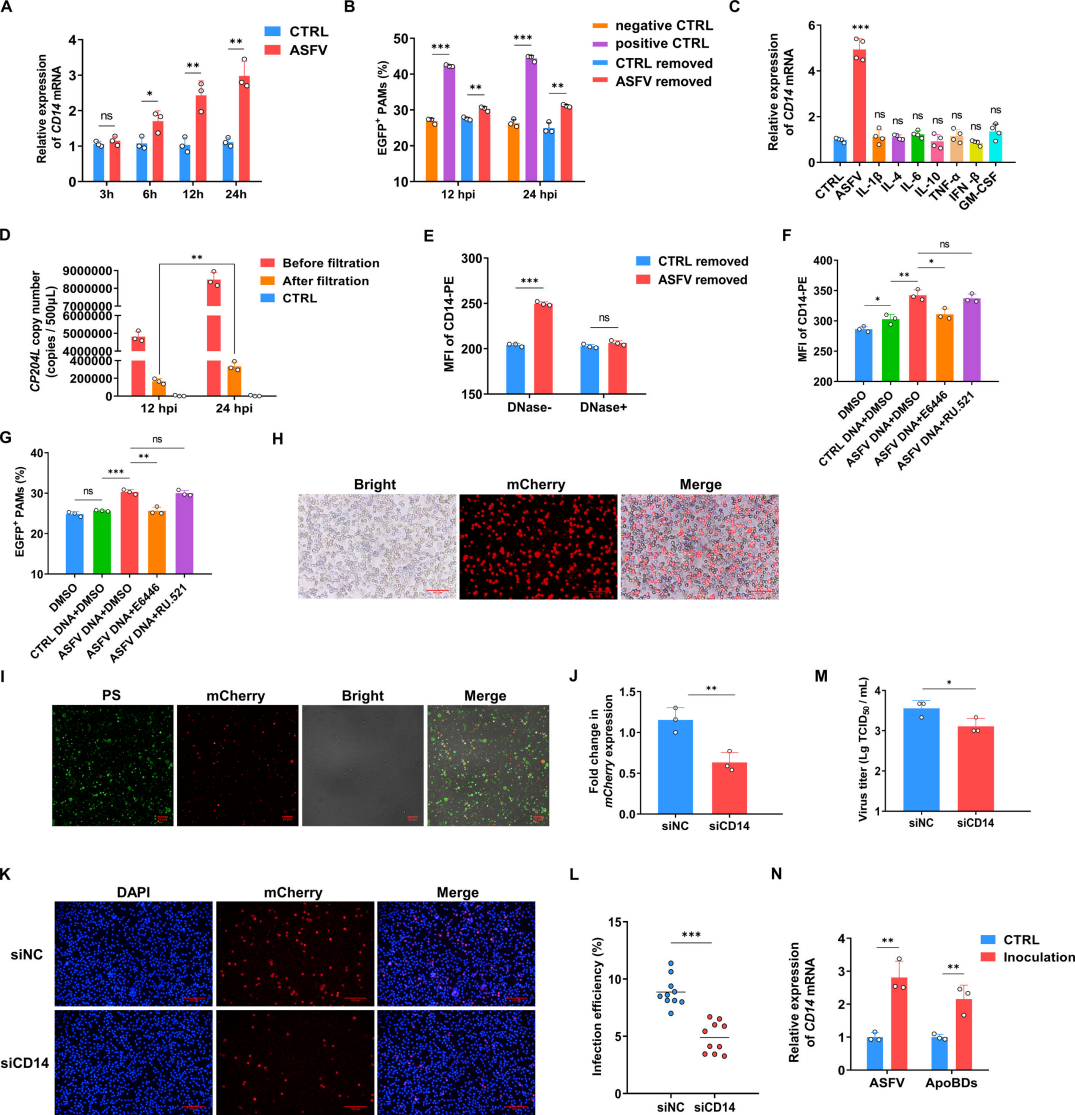
The upregulated CD14 in bystander PAMs facilitates viral transmission by enabling the phagocytosis of more ASFV-containing apoptotic bodies. (**A**) The supernatants from PAMs infected with ASFV (MOI 1) at 3, 6, 12, and 24 hpi were collected, filtered through a 0.1 µm PVDF filter to remove ASFV, and then added to untreated PAMs for culturing for 3 h. The relative level of *CD14* mRNA in PAMs was analyzed by qPCR (mean of three independent experiments ± SD). (**B**) The ASFV-removed supernatants from PAMs at 12 and 24 hpi were cultured with untreated PAMs for 3 h, respectively, and then infected with *E. coli*-EGFP (MOI 10) for 2 h. The “positive CTRL group” represents PAMs that were infected with unfiltered ASFV before bacterial infection. Flow cytometry was used to detect the phagocytic rate of PAMs (mean of three independent experiments ± SD). hpt, hours post-treatment. (**C**) PAMs were incubated with different cytokines at a concentration of 1 ng/mL (20 ng/mL for GM-CSF) for 3 h. The relative level of CD14 mRNA in PAMs was analyzed by qPCR (mean of four independent experiments ± SD). (**D**) The supernatants from PAMs infected with ASFV (MOI 1) at 12 and 24 hpi were collected, filtered through a 0.1 µm PVDF filter to remove ASFV, and extracted viral DNA. The level of *CP204L* was analyzed by qPCR, and the copy number was calculated using a standard curve (mean of three independent experiments ± SD). (**E**) PAMs were treated for 3 h with the ASFV-removed supernatant that was treated with DNase (4 U/100 µL) at 37°C for 30 min. The expression of CD14 was detected by flow cytometry (mean of three independent experiments ± SD). (**F, G**) PAMs were pre-treated with the TLR9 inhibitor E6446 (10 µM) for 1 h, and then with extracted viral DNA (24 hpi) for 3 h. The DNA extracted from the filtered supernatant of CTRL PAMs was used as the control. All groups were infected with *E. coli*-EGFP (MOI 10) for 2 h. CD14 MFI (**F**) and phagocytosis of *E. coli*-EGFP (**G**) were analyzed by flow cytometry (mean of three independent experiments ± SD). (**H**) PAMs were infected with ASFV-mCherry (MOI 2) for 24 hpi. ApoBDs were observed by IFA. Nuclei were stained with DAPI. Scale bar, 100 µm. (**I**) PAMs were infected with ASFV-mCherry (MOI 2) for 24 hpi. Isolated ApoBDs were observed by IFA. Phosphatidylserine (PS) was stained with Annexin V-Alexa 488 at room temperature for 15 min. The arrows point to only a subset of representative ApoBDs containing ASFV-mCherry. Scale bar, 25 µm. (**J**) PAMs were treated with siCD14 for 24 h, then incubated with ASFV-removed supernatant for another 3 h, and subsequently infected with ApoBDs-mCherry for 1 h. The DNA was extracted, and the level of *mCherry* was analyzed by qPCR (mean of three independent experiments ± SD). (**K**) PAMs were treated with siCD14 for 24 h, then incubated with ASFV-removed supernatant for another 3 h, and subsequently infected with ApoBDs-mCherry for 12 h. The infection efficiency of PAMs was observed by IFA. Nuclei were stained with DAPI. Representative data from multiple experiments are shown. Scale bar, 100 µm. (**L**) Calculate the percentage of infected PAMs (mean ± SD of 10 samples). (**M**) PAMs were treated with siCD14 for 24 h, then incubated with ASFV-removed supernatant for another 3 h, and subsequently infected with ApoBDs-mCherry for 12 h. The titer of ASFV in PAMs was detected using TCID_50_ (mean of three independent experiments ± SD). (**N**) The relative levels of *CD14* mRNA in PAMs at 3 hpi were analyzed by qPCR. The control inoculum was harvested from PAMs that had been induced to apoptosis by UV irradiation for 60 min. The supernatant and ApoBDs were isolated from it using the same method as the ASFV inoculum. The supernatant was used as the control for infection with ASFV particles, and the ApoBDs were used as the control for infection with ApoBDs (containing ASFV) (mean of three independent experiments ± SD).

As shown in Fig. 1, the phagocytic ability of bystander PAMs was also enhanced after ASFV treatment. To explore the mechanism of this enhancement, the effect of secretory factors on the bystander PAMs was evaluated. Using a 0.1 µm PVDF filter, the viral particles were removed from the cultured supernatant of ASFV-inoculated PAMs, collected at 3, 6, 12, and 24 hpi. The supernatant filtered at 24 hpi was re-inoculated onto PAMs for 24 h to confirm the absence of infectious ASFV particles in the filtrate obtained via a 0.1 µm PVDF membrane (Fig. S6E). Then, the filtrate was added to untreated PAMs to simulate the environment for bystanders (Fig. S7A). It was found that *CD14* mRNA level was upregulated after culturing with filtrate collected at 6, 12, and 24 hpi (Fig. 6A). Among them, the phagocytosis of PAM which were treated with filtrate collected at 12 or 24 hpi was further tested, and the enhancement of phagocytic ability was also observed (Fig. 6B). To further explore the reasons for CD14 upregulation in bystander PAMs, several cytokines that have been reported to potentially activate macrophage phagocytosis and mRNA levels, which were significantly increased after ASFV infection (Fig. S7B), were used to treat the PAMs and evaluate their effect on *CD14* transcription (43–48). However, the level of *CD14* mRNA in PAMs could not be effectively activated by adding them individually (Fig. 6C). To further investigate the potential phagocytic activation effects, TNF-α, which has the most related research on phagocytic activation, was used for phagocytosis experiments. It was discovered that neither low nor high levels of TNF-α can significantly enhance phagocytosis (Fig. S7C and E). TNF-α blocking experiment was also carried out, but it did not show any inhibitory effect on the phagocytosis enhanced by ASFV infection (Fig. S7D). It was considered whether the upregulation of CD14 in bystander PAMs could also be attributed to the stimulation of viral nucleic acids, similar to infected PAMs. To our surprise, despite the reduction after filtration, a significant number of viral DNA copies (*CP204L*) was still detected in the filtrate (Fig. 6D). The treatment with DNase degraded the viral nucleic acids within it (Fig. S7G) and prevented the upregulation of CD14 induced by the ASFV-removed supernatant (Fig. 6E). When viral DNA extracted from the filtered infected supernatant was added to untreated PAMs, it significantly increased CD14 expression and phagocytic ability, albeit to a small extent (Fig. 6F and G). Given that this viral DNA was not within intact viral particles, it was unlikely to undergo normal membrane fusion and escape from the endosome to the cytoplasm (49). Therefore, the cells were pretreated with E6446, an inhibitor of TLR9 which is a viral DNA sensor in the endosome (50), and found that it blocked CD14 upregulation, whereas cGAS inhibitors RU.521 had no significant effect (Fig. 6F and G). Thus, free viral DNA, but not secretory proteins, plays the role in inducing the upregulation of CD14 in bystander PAMs via TLR9 signaling.

### The upregulated CD14 in bystanders facilitates viral transmission by enhancing the phagocytosis of apoptotic bodies containing ASFV

In addition to phagocytizing bacteria, CD14 can also mediate the phagocytosis of apoptotic cells or ApoBDs by macrophages (51, 52). Our previous research has found ApoBD-dependent ASFV transmission (27). It raises a question of whether the upregulation of CD14 in bystander PAMs can increase the phagocytosis of ApoBDs, facilitating ASFV cell-to-cell transmission. Consistent with previous reports (27), we found ApoBDs containing mCherry in PAMs infected with ASFV-mCherry at 24 hpi (Fig. 6H). The ApoBDs were purified (Fig. 6I) as previously described (27). PAMs were pretreated with siRNA targeting CD14 for 24 h, then treated with ASFV-removed supernatant for 3 h to simulate bystander conditions, and finally, purified ApoBDs-mCherry were added (Fig. S7F). The relative level of mCherry in PAMs at 1 h post-phagocytosis was measured using qPCR to assess the phagocytosis of ASFV DNA in ApoBDs. The results showed that siCD14 treatment suppressed the upregulation of CD14 in PAMs induced by ASFV-removed supernatant, significantly reducing the phagocytosis of ApoBDs-mCherry (Fig. 6J; Fig. S7H). Furthermore, a significantly lower infection rate was observed in the siCD14-treated group compared to the siNC group at 12 h post-phagocytosis (Fig. 6K and L). Meanwhile, the viral titer also significantly decreased after siCD14 treatment (Fig. 6M). This indicates that the upregulation of CD14 in bystanders facilitates the phagocytosis of ApoBDs, which benefits viral transmission.

ApoBDs are generated progressively with ASFV infection. Our initial inoculum was harvested from ASFV-infected PAMs, which contained both ApoBDs (containing ASFV) and infectious ASFV particles. To determine whether ApoBDs (containing ASFV) in the inoculum contribute to the upregulation of CD14 in the early stages of infection, we isolated ApoBDs from the inoculum and infected PAMs with ApoBDs and ASFV particles at the same dose for 3 h. The results showed that the upregulation of CD14 in PAMs during the early stages of infection was induced jointly by ASFV and ApoBDs containing ASFV (Fig. 6N).

### Enhanced phagocytosis increases the possibility of a severe inflammatory response

**Fig 7:**
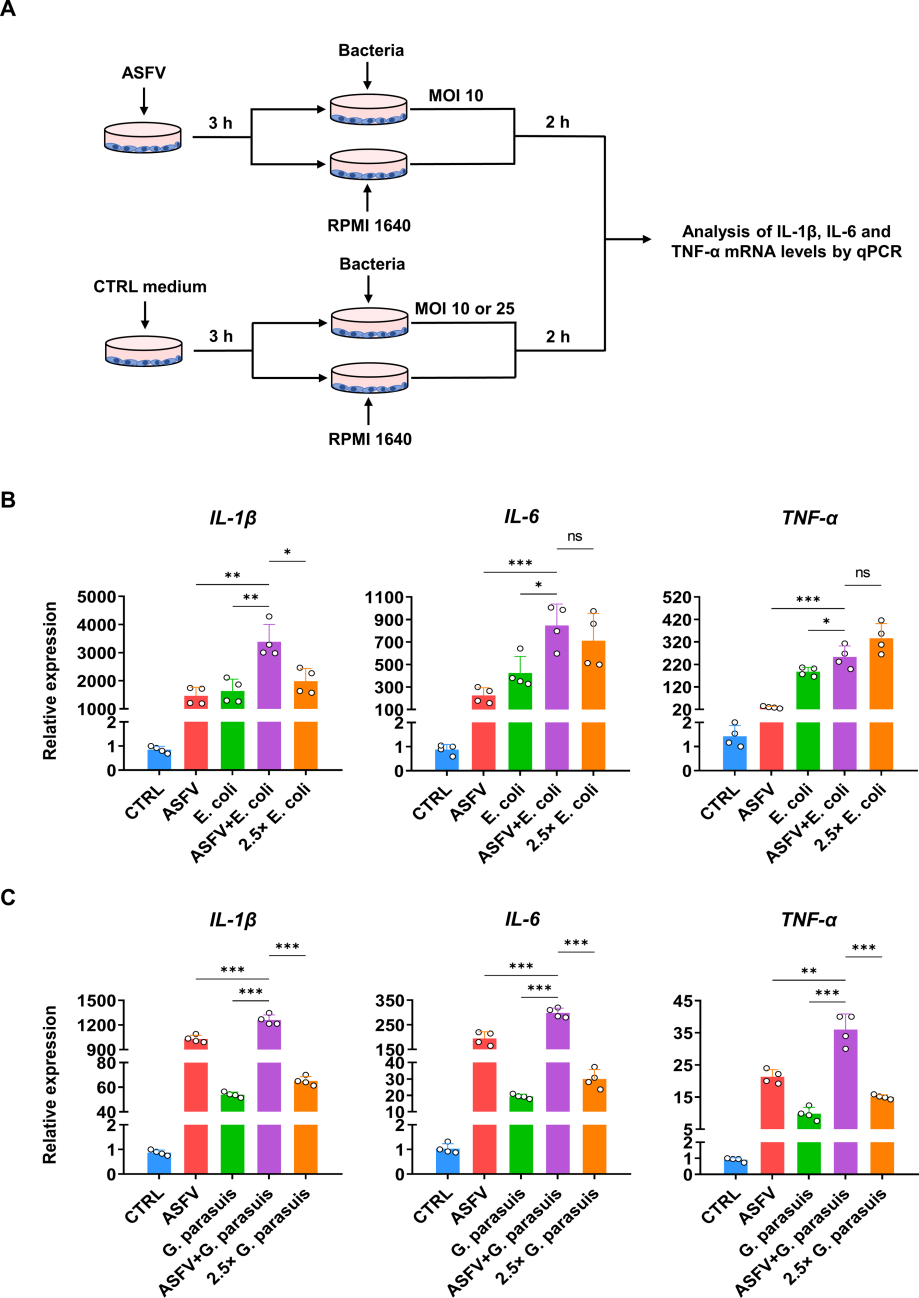
ASFV infection followed by exposure to bacteria increases the possibility of a stronger cytokine storm. (**A**) Experimental strategy for inflammatory cytokine detection. (**B**) PAMs were infected with ASFV for 3 h (MOI 1), then infected with *E. coli*-EGFP (MOI 10) for 2 h. The relative levels of *IL-1β*, *IL-6*, and *TNF-α* mRNA were analyzed by qPCR (mean of four independent experiments ± SD). (**C**) PAMs were infected with ASFV for 3 h (MOI 1), then infected with *G. parasuis* (MOI 25) for 2 h. The relative levels of *IL-1β*, *IL-6*, and *TNF-α* mRNA were analyzed by qPCR (mean of four independent experiments ± SD).

Since bacteria phagocytized by macrophages can cause a strong inflammatory response (53), it raises the question of whether the early stage of ASFV infection would lead to a more severe inflammatory response due to the enhanced phagocytosis in PAMs. The PAMs were inoculated with ASFV for 3 h and then infected with *E. coli*-EGFP at an MOI of 10 for 2 h. Then, qPCR was used to detect the mRNA levels of several major pro-inflammatory factors, including IL-1β, IL-6, and TNF-α. As ASFV infection leads to a higher bacterial phagocytic rate in PAMs, a control group of PAMs infected only with *E. coli* at an MOI of 25 was also established (Fig. 7A) to match the high phagocytic rate observed in ASFV-infected PAMs (Fig. S1E). The results showed that compared to PAMs infected with ASFV or *E. coli* alone, PAMs infected with ASFV and then with *E. coli* produced a more intense transcription of IL-1β, IL-6, and TNF-α (Fig. 7B). Compared to the group infected with high MOI bacteria alone, the coinfection group showed a more intense transcription of IL-1β (Fig. 7B). The same experiment was repeated with *G. parasuis,* and the transcription of IL-1β, IL-6, and TNF-α was also upregulated (Fig. 7C). In summary, these results indicate that the enhanced phagocytic ability of PAMs in the early stages of ASFV infection may contribute to a more intense cytokine storm-like phenomenon.

## DISCUSSION

Macrophages play a pivotal role in the innate immune system, with phagocytosis being one of the key functions. This process is fundamental for pathogen elimination, antigen presentation, and maintaining internal homeostasis (7). In the lungs, alveolar macrophages act as the first line of defense by detecting and engulfing inhaled pathogens or particles (18). As one of the major target cells, alveolar macrophages are easily infected by ASFV, impairing their ability to combat various pathogens (such as *Actinobacillus pleuropneumoniae* and *G. parasuis*). Previous studies have primarily focused on how ASFV suppresses innate immunity by regulating the interferon or apoptosis pathways in PAMs (54, 55). However, the effects of ASFV infection on the phagocytic activity of PAMs prior to cell death remain less explored (56). In the initial study, it was surprising to find that ASFV can increase the phagocytic ability of PAMs, which is the exact opposite of many reported pathogens (Fig. 1). Indeed, the phagocytic ability of ASFV-infected PAMs for fluorescent microspheres was significantly enhanced as well (Fig. S2G through I). Enhanced phagocytosis seems contradictory to the increased incidence of secondary bacterial infections that usually follow viral infections. However, the acidification of the endosomal system might be inhibited (34), and we are also researching it. This impairment may inadvertently allow the ingested bacteria to evade immune responses. Furthermore, the enhanced phagocytosis of PAMs can lead to the uptake of more bacteria and viruses, potentially resulting in more severe inflammatory responses and facilitating viral transmission.

The phagocytosis of macrophages is typically a synergistic process involving multiple receptors. Inhibiting one receptor may activate compensatory factors through alternative pathways to maintain stable phagocytic activity (33). In this study, CD14 was identified as a critical receptor in ASFV-enhanced phagocytosis. The knockdown of the other four receptors showed a limited inhibitory effect on phagocytosis, which is a plausible phenomenon, as these receptors may only have a limited role in the phagocytic capacity of ASFV-infected PAMs or their phagocytic function is compensated by alternative pathways. Once pathogens are recognized via specific receptors, signaling pathways are activated to initiate pseudopod extension, which is another crucial step in the phagocytic activity of macrophages. In a previous study, influenza virus was found to induce the formation of filopodia through CDC42 signaling, which results in enhanced virus endocytosis (57). To observe pseudopod dynamics, phalloidin was used to specifically label the functional filamentous actin (F-actin), and meanwhile, the activation of actin-regulating factors was also tested (58). Although F-actin was observed upon subsequent bacterial exposure, it is not activated by ASFV nor associated with enhanced phagocytosis in ASFV-infected PAMs.

CD14 is a glycosylphosphatidylinositol-anchored membrane protein without a China membrane domain, and it is primarily found on the surface of monocytes, macrophages, and dendritic cells (59). It serves as a receptor for bacterial PAMPs, such as LPS and LTA, binding to these molecules and facilitating their transfer to the TLR4-MD2 complex. This interaction initiates downstream signaling, which, in turn, triggers phagocytosis or the expression of inflammatory factors (60). Our research found that ASFV infection upregulates CD14 expression to enhance its binding to bacterial PAMPs, which can facilitate CD14-dependent phagocytosis of PAMs (Fig. 3F through H), but the level of TLR4 was not affected (Fig. S3D). We noticed that after siRNA knockdown of CD14, the PAMs are still able to phagocytize bacteria at a rate greater than the control group (Fig. 3G). It is likely that the efficiency of siRNA knockdown was affected due to the use of primary macrophages (PAMs) in this experiment, and as a result, the upregulation of CD14 induced by ASFV infection in PAMs was not completely inhibited by siRNA treatment. Initially, we suspected that some viral protein expressed in ASFV-infected cells might induce the increase of CD14, just as the Nef protein of human immunodeficiency virus (HIV) on human monocytes (61). However, the enhanced CD14-dependent phagocytosis was detected as early as 1 hpi (Fig. 4C and D), indicating it is an early event during ASFV infection. Also, the heat-inactivated ASFV induced a certain degree of CD14 upregulation, further proving this phenomenon is unrelated to viral replication (Fig. 4B). The upstream signals stimulating CD14 transcription are complex, as pathogen infection may enhance the transcription of CD14 by activating NF-κB, MAPKs, or AP-1 (62, 63). Considering the complexity of the upstream signals, the core downstream transcription factors were first studied and ultimately determined that the translocation of NF-κB p65 is essential for the upregulation of CD14 in ASFV-infected PAMs (Fig. 4K). However, the activation of NF-κB is fundamental for cells to perform a variety of functions (64). It remains to be further investigated whether it directly upregulates CD14 transcription or does so indirectly through other transcription factors following ASFV infection. As the heat-inactivated ASFV can also induce the upregulation, the internalized viral nucleic acids and matrix proteins might be the trigger. So the intracellular DNA sensor cGAS, particularly recognizing pathogenic dsDNA, was tested (65). And it was found that inhibiting the signal transduction of cGAS or STING greatly suppressed the upregulation of CD14 and phagocytic ability induced by ASFV (Fig. 5F and G). But a little of p65 translocation can still be found after the cGAS/STING pathway is blocked. This suggests that inflammatory factors or other components in the viral suspension may also stimulate the nuclear translocation of NF-κB, but this is not related to the upregulation of CD14 (Fig. 6C and E). Different upstream stimuli can lead to different post-translational modifications of p65, and these modifications affect its ability to bind to the promoters of different genes after nuclear translocation (66, 67). As a large dsDNA virus with a genome around 150 kb and 150 nm virion, PRV is also recognized by cGAS-STING (68), but it cannot induce the increased CD14 expression and phagocytosis in PAMs (Fig. S2E, F, S4D and S6C), indicating the differences in the downstream activation between ASFV and PRV (42, 68, 69). Since the upregulation of CD14 is an early event after ASFV infection, the cGAS-STING or NF-κB signaling has not been antagonized by expressed viral protein in PAMs yet (70–73).

Except for the ASFV-infected PAMs, the bystander cells in the inoculation group also showed enhanced phagocytic ability with relatively lower levels. However, it is worth noting that the phagocytic ability of bystander cells increases with the duration of infection (Fig. 1A; Fig. S2A), strongly suggesting that infected PAMs may secrete some factor responsible for this phenomenon. Considering the phagocytic function of macrophages is easily regulated by various cytokines in the environment (33), several phagocytosis-related inflammatory cytokines with upregulated transcription during ASFV infection were tested to see whether they can stimulate the transcription of CD14 in PAMs (43–48). But neither added cytokines nor antagonists were found to change the expression of CD14. Previous studies have examined the impact of IL-6, TNF-α, and GM-CSF on CD14 transcription in macrophages, revealing that even at high doses significantly more than those induced by ASFV infection (74–77), it was challenging to achieve a significant increase within 12 h of treatment (38, 78, 79). This suggests that there may be other reasons contributing to the CD14 upregulation in bystander PAMs in this study. It was found that the ASFV-removed filtrate still contained significant amounts of viral nucleic acids released from ASFV-infected cells (Fig. 6D). These nucleic acids were easily internalized by nearby PAMs (80) and ultimately degraded through the endosome-lysosome pathway, rather than escaping from the endosomes as the virus infection (49, 81). During this process, the DNA can be recognized by TLR9, which is located in endosomes and lysosomes (50). TLR9 specifically recognizes unmethylated CpG-DNA, a structure commonly found in some bacteria or viruses, including ASFV (82). The classical pathway of TLR9 signaling also works through NF-κB to regulate the transcription of various genes (26), which may explain why the upregulation of CD14 was completely blocked by NF-κB antagonists. Due to the continuous death and release of contents (including free ASFV DNA) from infected cells in the later stages of ASFV infection, the stimulation of bystander cells is also ongoing. This may explain why the upregulation of bystander CD14 detected under infection conditions is stronger than that induced by incubating with infected supernatant or by exogenously adding ASFV DNA (Fig. 3I; Fig. 6B and F). CD14 also plays an important role in the phagocytosis of ApoBDs, and its upregulation can significantly promote phagocytosis (51, 52). Our recent research found that ASFV can transmit through ApoBDs, which is one of the main modes of ASFV transmission (27). This study further demonstrates that ASFV infection helps PAMs in phagocytizing ApoBDs through enhancing CD14-dependent phagocytosis, thereby accelerating the transmission of ASFV (Fig. 6J through M). We noticed that the knockdown of CD14 did not affect the viral titer of ASFV directly infected PAMs (Fig. S4A). In addition to transmission via ApoBDs, ASFV can also transmit through other ways, such as release (27), which may have masked the observation of the function of CD14 in ASFV transmission in this experiment (Fig. S4A). However, the transmission of ASFV via ApoBDs is a way of transmission that can evade neutralizing antibodies (27) and has greater clinical significance. Although the upregulation of CD14 induced by ASFV infection appears to be predictable, the discovery of its role in viral transmission is meaningful.

Usually, viral infection can create conditions for secondary bacterial infection and aggravate the harm of infection. For example, PRRSV, influenza virus, and SARS-CoV-2 can easily lead to secondary bacterial infections, resulting in increased mortality primarily due to co-infections rather than the virus alone (83–85). Some viruses achieve this by suppressing the function of macrophages, while others damage the mucosal barrier (8, 86). However, there is still limited research on ASFV enhancing the phagocytic ability in the early stages of viral infections to facilitate bacterial infections or cause more damage. To further investigate the impact of this increased phagocytosis, the transcription of pro-inflammatory factors was analyzed, with bacteria inoculation at the early stages of ASFV infection. It was noticed that the activation is generally higher than the sum of individual stimulating effects from ASFV and bacteria, higher than the effect of bacteria at 2.5 times MOI of inoculation as well. This increases the risk of triggering more intense cytokine storm-like symptoms and causing more severe lung lesions. In addition, the impaired killing ability may facilitate the dissemination of bacteria *in vivo*, which we are currently investigating.

**Fig 8:**
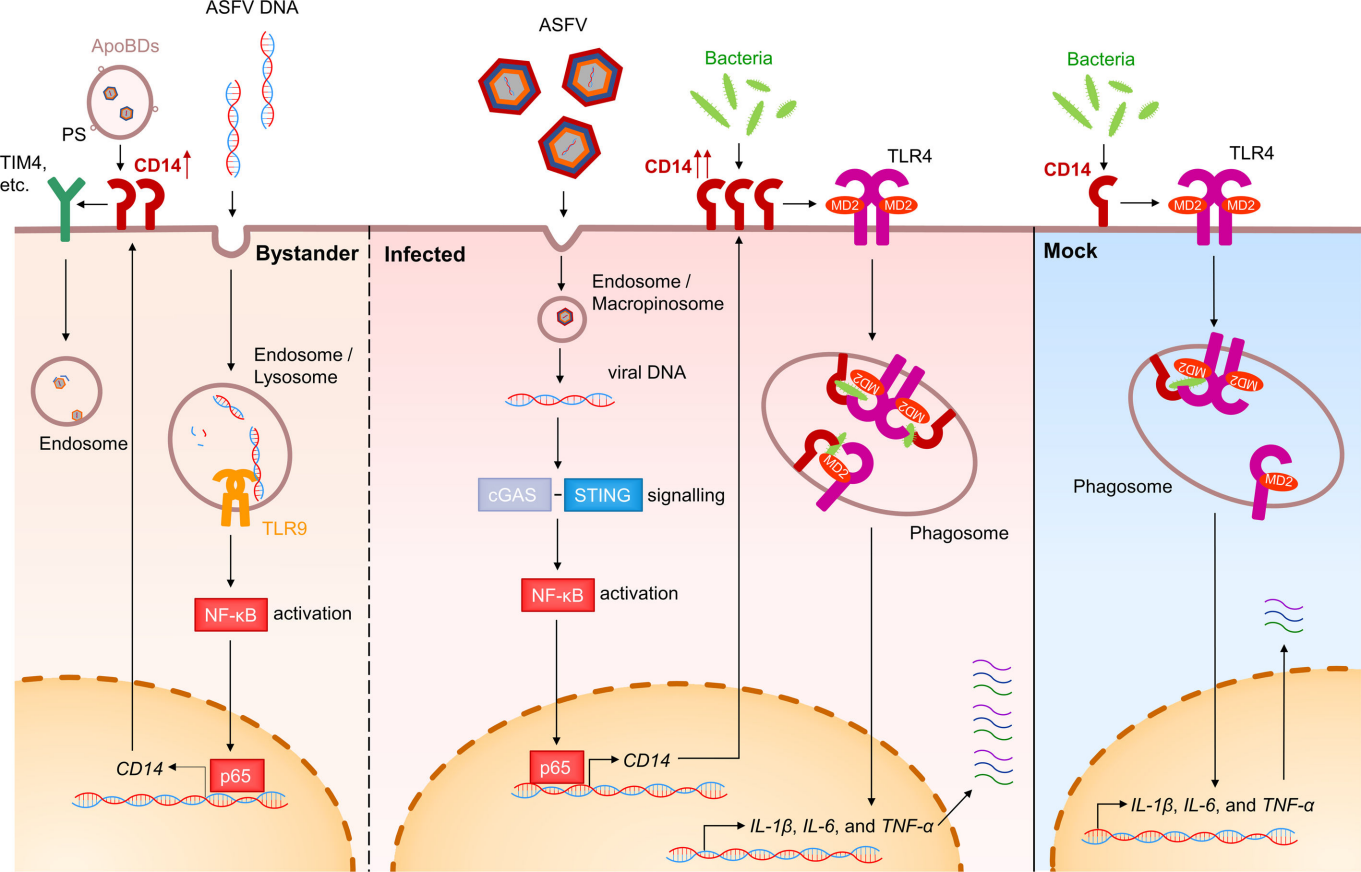
Model of ASFV infection enhances the phagocytosis of PAMs. In the early stages of ASFV internalization, the viral dsDNA released into the cytoplasm is recognized by the cGAS-STING pathway, inducing the downstream nuclear translocation of NF-κB p65. This ultimately activates the transcription and expression of CD14 in PAMs. Free viral DNA released from infected PAMs enters bystander PAMs and enhances CD14 expression via the TLR9 pathway. The high expression of CD14 on the cell membrane surface facilitates phagocytosis in PAMs and induces a stronger transcription of pro-inflammatory cytokines (IL-1β, IL-6, and TNF-α). The upregulation of CD14 in bystanders enhances the phagocytosis of ApoBDs-containing ASFV, which benefits the transmission of the virus.

In summary, the impact and significance of ASFV infection on the phagocytic process of PAMs were investigated in this study. It was discovered that phagocytosis can be significantly enhanced after ASFV infection, and the cGAS-STING pathway is activated by viral DNA to induce the nuclear translocation of NF-κB p65, which, in turn, further activates the expression of CD14. Meanwhile, released ASFV nucleic acid from infected PAMs can also enhance CD14 expression in bystanders through the TLR9 pathway. Previous studies have mainly focused on how the innate immune pathways were antagonized by ASFV (87–89), but here we identified two pathways by which ASFV enhances CD14-dependent phagocytosis. Especially, the CD14 upregulation in bystander PAMs can promote viral transmission via ApoBDs. Moreover, the enhanced phagocytosis may lead to a stronger inflammatory response after bacterial exposure, which is also of concern (Fig. 8). These findings provide new insights into the interaction between ASFV and the host immune system.

## MATERIALS AND METHODS

### Cells and virus

Primary porcine alveolar macrophages (PAMs) were collected from 1-month-old SPF pig (Large White) (Beijing Center for SPF Swine Breeding & Management) as previously described (90) and grown in RPMI 1640 medium (Gibco, Waltham, MA, USA) containing 10% fetal bovine serum (FBS) (Gibco, Rockville, MD, USA), which was reduced to a 2% concentration during viral infection, at 37℃ in a 5% CO_2_ atmosphere. Genotype II ASFV strain CADC_HN09 (GenBank accession number MZ614662.1) and the fluorescence-labeled virus HN09-mCherry with mCherry to replace its CD2v gene were cultured in PAMs, with a titer of 10^6.5^ TCID_50_/mL. The HP-PRRSV strain JXwn06 (GenBank accession number EF641008) with a titer of 10^6.5^ TCID_50_/mL and PRV strain HB1201 (GenBank accession number KU057086.1) with a titer of 10^7^ TCID_50_/mL were used in this study. All virus solutions were centrifuged at 10,000 × *g* for 5 min to remove cellular debris. All experimental results were validated in PAMs from more than three pigs. All experiments involving ASFV infection were conducted in the biosafety level 3 laboratory of China Agricultural University in strict accordance with the relevant biosafety regulations and operating procedures (approval no. 2022-ASFV-002).

### Main reagents and antibodies

NF-κB inhibitor BAY 11-7082 (HY-13453), cGAS inhibitor RU.521 (HY-114180), STING inhibitor C176 (HY-112906), TLR9 inhibitor E6446 (HY-12756), and bacterial adhesion inhibitor Sibofimloc (HY-12820) were purchased from MedChemExpress (Monmouth Junction, NJ, USA). The murine monoclonal antibodies against ASFV p30 or PRRSV N protein, and rabbit polyclonal antibodies against PRV GP5 protein, were prepared by our laboratory. PE-conjugated CD14 murine monoclonal antibody (ab186689) and Alexa Fluor 568-conjugated goat anti-rabbit IgG antibody (ab175471) were purchased from Abcam (Cambridge, UK). MYH9 (PA5-29673) rabbit polyclonal antibody, Alexa Flour 488-conjugated goat anti-mouse IgG antibody (A-11001), Alexa Flour 647-conjugated goat anti-mouse IgG antibody (A-21235), and Alexa Flour 647-conjugated goat anti-rabbit IgG antibody (A-21244) were purchased from Invitrogen (Carlsbad, CA, USA). Alexa Flour 594-conjugated Phalloidin (C2205S) was purchased from Beyotime Biotechnology (Jiangsu, China). Annexin V-Alexa Fluor 488 (CA1040) was purchased from Solarbio Life Sciences (Beijing, China). RNase-Free DNase (M6101) was purchased from Promega (Madison, WI, USA).

### Virus infection

Primary PAMs were infected with ASFV at an MOI of 0.5, 1, or 2. Incubation with the thawed suspension of negative PAMs was used as a control. After incubation at 37°C for 1 h, the unbound viruses were removed by washing with PBS. The medium was replaced with RPMI 1640 containing 2% FBS for further study. The infection methods for PRRSV and PRV are the same as those for ASFV.

### DNA/RNA extraction and qPCR

ASFV genomic DNA was isolated from PAMs using the TIANamp Genomic DNA kit (Tiangen, Beijing, China). Total RNA was extracted using TRIzol reagent (Thermo Fisher Scientific, Waltham, MA, USA), and reverse transcription was accomplished with the FastKing RT Kit (Tiangen, Beijing, China). The DNA and cDNA were amplified using the BioRad CFX96 Real-Time PCR system with UltraSYBR Mixture (CWBIO, Jiangsu, China) according to the manufacturer’s instructions. Primer sequences for qPCR are listed in Table S1. The data were normalized based on the level of β-actin in each sample. All experiments were independently conducted in triplicate at least.

### Construction of the EGFP-labeled *E. coli*

The EGFP gene was amplified from the plasmid pEGFP-N2 using primers that contained the EcoR I and Hind III restriction sites, respectively, and then cloned into plasmid pET-28a. The recombinant plasmid was transformed into DH5α competent cells for sequencing. And the confirmed plasmid was transformed into BL21-competent cells to construct the *E. coli*-EGFP for further use.

### Bacterial culture and plate colony counting

*E. coli*-EGFP was grown in 1 mL of Luria-Bertani (LB) broth at 37℃ in a shaking incubator. It was collected at different OD_600_ values (38, 0.65, 1.03, 1.25, and 1.34) as determined spectrophotometrically, and then cultured them through serial dilution on LB agar plates in a 37℃ incubator overnight to calculate the bacterial growth curve (OD_600_-CFU). For the phagocytosis assay, *E. coli*-EGFP was grown in LB broth at 37℃ in a shaking incubator. IPTG was added at an OD_600_ of 0.6, and then it was cultured for 3 h. Afterward, the OD_600_ was adjusted to 1.03 (7 × 10^7^ CFU/mL) for further study.

*Glaesserella parasuis* serotype 13 was isolated and preserved by our laboratory. It was grown in 1 mL of LB broth supplemented with 5% FBS and 0.1% nicotinamide adenine dinucleotide (NAD) (Solarbio, Beijing, China). The determination of the bacterial growth curve was the same as mentioned above.

### Labeling of *G. parasuis* with FITC

The overnight culture of *G. parasuis* was washed and resuspended in PBS to a final concentration of 2 × 10^8^ CFU/mL. The bacterial pellet was then suspended in 1 mL of PBS containing 10 mM FITC (Solarbio, Beijing, China) and incubated in a shaking incubator at 37°C for 1 hour. Subsequently, the bacteria were washed three times with PBS to remove any unbound FITC, preparing them for further analysis.

### Phagocytosis assay

Primary PAMs were seeded in 24-well plates (1 × 10^6^ cells/well). After different treatments, PAMs were washed with PBS and then mixed with *E. coli*-EGFP at a MOI of 10 in RPMI 1640 medium containing 5% FBS. The phagocytosis assays involving *G. parasuis* and beads-FITC (L4655, Sigma, St. Louis, MO, USA) were similar to that with *E. coli*-EGFP, except that *G. parasuis* was used at an MOI of 25, while the beads-FITC assay was performed at a concentration of 1 × 10^8^ cells/well. All groups were cultured at 37℃ in a 5% CO_2_ atmosphere for 2 h (1 h for beads). Then the PAMs were washed twice with PBS to remove the bacteria or beads outside.

### Plate colony counting the intracellular *G. parasuis* phagocytized by PAMs

After the phagocytosis of *G. parasuis* was completed, the supernatant was discarded, and the PAMs were washed twice with 0.5 mL of PBS. Subsequently, 0.2 mL of PBS containing 200 μg/mL gentamicin was added to each well and incubated for 20 min to kill extracellular bacteria (91). The supernatant was then discarded, and each well was washed twice with 0.5 mL of PBS. Next, 0.5 mL of sterile water was added to lyse the PAMs and release intracellular bacteria. All samples were serially diluted and cultured on LB agar plates containing 5% FBS and 0.1% NAD in a 37°C incubator for 16 h. The bacterial CFU in the original samples was calculated based on the dilution factors.

### siRNA treatment

The targeting sequences of siRNAs were synthesized by GenePharma (Jiangsu, China). All sequences are listed in Table S2. Primary PAMs were transfected with 20 nM siRNA or siNC using Lipofectamine RNAiMAX (Thermo Fisher Scientific, Waltham, MA, USA) according to the manufacturer’s instructions. After 24 h, the transfected PAMs were infected with ASFV at an MOI of 0.5. At 24 hpi, the cells were used to perform a phagocytosis assay or flow cytometry.

### Surface/intracellular staining and flow cytometry

The treated PAMs were resuspended in 2% paraformaldehyde (PFA) solution on ice for 15 min. After being washed with PBS, the cells were incubated in PBS containing 10% mouse serum on ice for 15 min to block non-specific binding. Surface staining was performed for 30 min with the fluorescently labeled antibodies. For intracellular staining, the cells were resuspended in 0.1% Triton X-100 on ice for 10 min. After being washed with PBS, the cells were sequentially stained with primary and fluorescently labeled secondary antibodies for 30 min each. Surface staining was performed after intracellular staining when the primary antibodies were from the same species.

Flow cytometric analysis was performed (at least 10,000 cells/condition) on FACS Canto II instruments (BD Biosciences), and the data were analyzed using FlowJo software (Tree Star Inc.). All experiments were conducted at least in triplicate.

### Immunofluorescence microscopy

Primary PAMs, seeded on glass coverslips, were treated differently for the indicated times, washed with PBS, and fixed with 4% PFA for 15 min at room temperature (RT). The cells were permeabilized with 0.1% Triton X-100 for 10 min at RT, then washed three times with PBS and blocked with PBS containing 2% bovine serum albumin (BSA) for 15 min. The permeabilized cells were sequentially incubated with primary and fluorescently labeled secondary antibodies for 1 h at RT. Cell nuclei were stained with 4′, 6-diamino-2-phenylindole (DAPI) for 7 min at RT. Coverslips were mounted with Aqua-Poly/Mount (Polysciences, Warrington, PA, USA) on microscope slides. Images were observed and recorded with a Nikon A1 confocal microscope.

For cells that did not need antibody staining, they were directly observed and recorded using the Eclipse Ti2 microscope (Nikon). Counting was conducted in at least eight different fields of view along the diagonal direction.

### Cell viability assay

Primary PAMs were seeded in a 96-well plate and cultured with RPMI 1640 medium containing 2% FBS and different concentrations of BAY 11-7082, RU.521 or C176 for 1 h at 37℃ in a 5% CO_2_ atmosphere. DMSO was used as a control. Then, 10 µL of CCK-8 solution (BMU106, Abbkine, Wuhan, China) was added, and the culture was continued for 1 h. Finally, a TECAN SPARK microplate reader (Groedig, Austria) was used to detect the absorbance at 450 nm and calculate the cell viability. All experiments were conducted in triplicate.

### Statistical analysis

GraphPad Prism 10.1.2 software was used to perform statistical analysis. The difference between different groups was analyzed by a two-tailed Student’s t-test. The data are expressed as the mean ± standard deviation (SD). The star * indicates a significant difference (*p* < 0.05), ** a highly significant difference (*p* < 0.01), and *** an extremely significant difference (*p* < 0.001). All experiments were performed in triplicate (*n* = 3) at least.

## Data Availability

All data supporting the findings in the paper are available within the paper and its supplemental material. All relevant data are available from the authors.

## SUPPLEMENTAL MATERIAL

Fig. S1 jvi.00690-25-s0001.tiff Construction of EGFP-labeled *E. coli* and phagocytic model.

Fig. S2 jvi.00690-25-s0002.tiff ASFV infection enhances the phagocytosis of PAMs.

Fig. S3 jvi.00690-25-s0003.tiff ASFV infection does not affect the migration, bacterial adhesion, and pseudopod extension abilities of PAMs.

Fig. S4 jvi.00690-25-s0004.TIF ASFV infection enhances the phagocytosis of PAMs by upregulating the expression of CD14.

Fig. S5 jvi.00690-25-s0005.TIF The enhancement of CD14-dependent phagocytosis by ASFV is dependent on NF-κB activation.

Fig. S6 jvi.00690-25-s0006.TIF ASFV enhances the CD14-dependent phagocytosis through cGAS/STING/NF-κB pathways.

Fig. S7 jvi.00690-25-s0007.TIF Free ASFV DNA in the supernatant can enhance the CD14 expression in bystander PAMs.

Supplemental legends jvi.00690-25-s0008.pdf Legends for Fig. S1 to S7.

Table S1 jvi.00690-25-s0009.docx Primer sequences for qPCR.

Table S2 jvi.00690-25-s0010.docx siRNA sequences.

ASM does not own the copyrights to Supplemental Material that may be linked to, or accessed through, an article. The authors have granted ASM a non-exclusive, world-wide license to publish the Supplemental Material files. Please contact the corresponding author directly for reuse.
