# Supplementary material for: African swine fever virus infection enhances CD14-dependent phagocytosis of porcine alveolar macrophages to promote bacterial uptake and apoptotic body-mediated viral transmission
Source: J Virol. 2025 Jun 12;99(7):e00690-25. doi: 10.1128/jvi.00690-25 (PMC12282190; doi:10.1128/jvi.00690-25)
Supplement: Supplemental legends — Legends for Fig. S1 to S7. [file jvi.00690-25-s0008.pdf]

Supplemental Figure Legends

Figure S1. Construction of EGFP-labeled *E. coli* and phagocytic model. (A) The pET-28a-EGFP plasmid was transformed into *E. coli* (BL21). A distinct green fluorescence was observed after IPTG induction. Scale bars, 50 μm. (B) Determination of the OD600-CFU (colony forming unit) curve for the *E. coli*-EGFP. (C) Images showing PAMs phagocytizing *E. coli*-EGFP (MOI 25) at different FBS concentrations. Scale bars, 20 μm. (D) Flow cytometry scatter plots of PAMs phagocytizing *E. coli*-EGFP (MOI 25) at different FBS concentrations. (E) Flow cytometry scatter plots of PAMs phagocytizing *E. coli*-EGFP at different MOIs (bacteria: PAMs). Representative data from multiple experiments are shown. (F) The percentage of PAMs phagocytizing *E. coli*-EGFP at different MOIs (bacteria: PAMs) (mean of three independent experiments ± standard deviation (SD). (G) Flow cytometry scatter plots of PAMs phagocytizing *E. coli*-EGFP at different time points post inoculation. Representative data from multiple experiments are shown. (H) The percentage of PAMs phagocytizing *E. coli*-EGFP at different time points post inoculation (mean of three independent experiments ± SD).

Figure S2. ASFV infection enhances the phagocytosis of PAMs. (A) Flow cytometry scatter plots of PAMs phagocytizing *E. coli*-EGFP (MOI 10) at 12 h after ASFV infection (MOI 0.5). Representative data from multiple experiments are shown. (B) The percentage and MFI of PAMs phagocytizing *E. coli*-EGFP (MOI 10) at 24 h after ASFV infection (MOI 0.5) (mean of three independent experiments ± SD). (C) The percentage and MFI of infected and bystander PAMs phagocytizing E. coli-EGFP (MOI 10) at 12 h after ASFV infection (MOI 0.5). n=3. (D) Colony plate of PAMs phagocytizing *G. parasuis* (MOI 25) at 24 h after ASFV infection (MOI 0.5). (E) Flow cytometry scatter plots of PAMs phagocytizing *E. coli*-EGFP (MOI 10) at 24 h after PRV infection (MOI 0.5). Representative data from multiple experiments are shown. (F) The percentage and MFI of PAMs phagocytizing *E. coli*-EGFP (MOI 10) at 24 h after PRV infection (MOI 0.5) (mean of three independent experiments ± SD). (G) Flow cytometry scatter plots of PAMs phagocytizing beads-FITC at 24 h after ASFV infection (MOI 0.5). Representative data from multiple experiments are shown. (H) The percentage and MFI of PAMs phagocytizing beads-FITC at 24 h after ASFV infection (MOI 0.5) (mean of three independent experiments ± SD). (I) IFA analysis of PAMs phagocytizing beads-FITC at 24 h after ASFV infection (MOI 0.5). Scale bars, 10 μm.

Figure S3. ASFV infection does not affect the migration, bacterial adhesion, and pseudopod extension abilities of PAMs. (A) PAMs were infected with ASFV for 24 h (MOI 0.5), and then infected with *E. coli*-EGFP (MOI 10) for 2 h in an environment containing the FimH inhibitor Sibofimloc. The phagocytosis was observed under a fluorescence microscope. Representative data from multiple experiments are shown. Scale bars, 50 μm. (B) Calculate the percentage of phagocytosis (mean of three independent experiments ± SD). (C) IFA analysis of pseudopod extension at 24 h post ASFV infection (MOI 0.5). F-actin were labeled with Alexa 594-conjugated Phalloidin. ASFV-infected PAMs were labeled with an antibody against p30 followed by an Alexa 488-conjugated secondary antibody. Representative data from multiple experiments are shown. Scale bars, 10 μm. (D) The relative levels of *TLR4*, *CD163*, *COLEC12* and *CD169* mRNA after ASFV infection (MOI 0.5) were analyzed by qPCR (mean of three independent experiments ± SD).

Figure S4. ASFV infection enhances the phagocytosis of PAMs by upregulating the expression of CD14. (A-B) Results of the viral titer (A) in the whole cell lysate and CCK8 assay (B) of PAMs after different siRNAs treatment for 24 h, followed by ASFV infection for an additional 24 h (mean of three independent experiments ± SD). (C) MFI of CD14 expression in PAMs at 24 hpi after PRRSV infection (mean of three independent experiments ± SD). (D) MFI of CD14 expression in PAMs at 24 hpi after PRV infection (mean of three independent experiments ± SD). (E) Following ultrafiltration of supernatant from ASFV-infected PAMs through a 100 kDa molecular weight cutoff tube, the filtrate incubated with untreated PAMs for 1 h, and then cultured for 24 h to assess for ASFV infection. ASFV infected PAMs were labeled with an antibody against p30 followed by an Alexa 488-conjugated secondary antibody. Representative data from multiple experiments are shown. Scale bars, 100 μm.

Figure S5. The enhancement of CD14-dependent phagocytosis by ASFV is dependent on NF-κB activation. (A) The relative levels of CD14 mRNA after ASFV infection (MOI 1) was analyzed by qPCR (mean of three independent experiments ± SD). "0 min" indicates that ASFV was pre-adsorbed to PAMs for 30 min at 4 °C. (B) PAMs were pre-treated with various concentrations of BAY 11-7082 for 1 h, and then cultured for 3 h. The cell viability of PAMs was assessed using the CCK8 assay (mean of three independent experiments ± SD). (C) PAMs were pre-treated with different concentrations of BAY 11-7082 for 1 h, and then infected with ASFV for 1 h (MOI 1). The cells were collected after an acid wash, and extracted viral DNA. The level of *CP204L* was analyzed by qPCR (mean of three independent experiments ± SD).

Figure S6. ASFV enhances the CD14-dependent phagocytosis through cGAS/STING/NF-κB pathways. (A) PAMs were pre-treated with various concentrations of RU.521 or C176 for 1 h, and then cultured for 3 h. The cell viability of PAMs was assessed using the CCK8 assay (mean of three independent experiments ± SD). (B) PAMs were pre-treated with RU.521 (20 μM) or C176 (20 μM) for 1 h, and then infected with ASFV for 1 h (MOI 1). The cells were collected after an acid wash, and extracted viral DNA. The level of *CP204L* was analyzed by qPCR (mean of three independent experiments ± SD). (C) Flow cytometry scatter plots of PAMs phagocytizing *E. coli*-EGFP (MOI 10) after infection with PRV for 3 h (MOI 1). Representative data from multiple experiments are shown. (D) MFI and phagocytic rate of *E. coli*-EGFP (MOI 10) in PAMs 3 h after PRV infection (mean of three independent experiments ± SD). (E) Following filtering of the supernatant from ASFV-infected PAMs at 24 hpi through a 0.1 μm PVDF membrane, the filtrate was incubated with untreated PAMs for 1 h, and then cultured for 24 h to assess for ASFV infection. ASFV infected PAMs were labeled with an antibody against p30 followed by an Alexa 488-conjugated secondary antibody. Scale bars, 100 μm.

Figure S7. Free ASFV DNA in the supernatant can enhance the CD14 expression in bystander PAMs. (A) Experimental strategy to simulate the phagocytosis of bystander PAMs. (B) The relative levels of *IL-1β*, *IL-4*, *IL-6*, *IL-10*, *TNF-α*, *IFN-β* and *GM-CSF* mRNA in PAMs after ASFV infection (MOI 1) were analyzed by qPCR (mean of three independent experiments ± SD). (C) Flow cytometry scatter plots of PAMs phagocytizing *E. coli*-EGFP (MOI 10) after the addition of TNF-α. Representative data from multiple experiments are shown. (D) PAMs were infected with ASFV for 24 h (MOI 0.5) in the presence of a TNF-α antibody blockade (20 μg/mL), and then infected with *E. coli*-EGFP (MOI 10) for 2 h. Flow cytometry was used to detect the phagocytic rate of *E. coli*-EGFP. Representative data from multiple experiments are shown. (E) The phagocytic rate of PAMs on *E. coli*-EGFP (MOI 10) after the addition of TNF-α (mean of three independent experiments ± SD). (F) Experimental strategy to simulate the phagocytosis of ApoBDs containing ASFV by bystander PAMs. (G) After treatment with DNase (4 U/100 μL) at 37°C for 30 min, the copy number of *CP204L* in the ASFV-removed supernatant was determined by qPCR (mean of three independent experiments ± SD). (H) PAMs were treated with siCD14 for 24 h, then incubated with ASFV-removed supernatant for another 3 h. The relative level of *CD14* mRNA was analyzed by qPCR (mean of three independent experiments ± SD).
