# Supplementary material for: African swine fever virus infection enhances CD14-dependent phagocytosis of porcine alveolar macrophages to promote bacterial uptake and apoptotic body-mediated viral transmission
Source: J Virol. 2025 Jun 12;99(7):e00690-25. doi: 10.1128/jvi.00690-25 (PMC12282190; doi:10.1128/jvi.00690-25)
Supplement: Table S1 — Primer sequences for qPCR. [file jvi.00690-25-s0009.docx]

**Table S1. Primer sequences for qPCR**

| **Gene** | **Sequences 5’ to 3’** |
| --- | --- |
| TLR2 | F: TGCTATGACGCTTTCGTGTC  R: TTCTCGGAAAGCACGAAGAT |
| CD14 | F: CCTGCATGCGCTCGGGTTCTC  R: GCCCGGGATTGTCAGATAGGTC |
| CD48 | F: CCAAGTCAGCAATCCTGTGA  R: AGGAGGCTAAGAACGGTGGT |
| CD64 | F: GCCACAGAAGATGGAAAG  R: CTACAGGCACCCACAGAG |
| SDC4 | F: CACCGAACCCAAGGAACT  R: AGCAAAGAGGATGCCCAC |
| TLR4 | F: ATATGGCAGAGGTGAAAGCAC  R: GAAGGCAGAGATGAAAAGGGG |
| CD163 | F: ATTCATCATCCTCGGACCCAT  R: CCCAGCACAACGACCACCT |
| COLEC12 | F: TCCGTTTGGATTCTGTTT  R: GTCCTCTGTCACCTTTCG |
| RAC1 | F: ACACGACCAATGCCTTTC  R: ATGGTGTCGCACTTCTGG |
| RHOA | F: AGGTAGAGTTGGCTTTGTG  R: TTTACTGGCTCCTGCTTC |
| CDC42 | F: GGGCAAGAGGATTATGAC  R: TCCCAACGAGCAAGAAAG |
| IL-4 | F: CCAACCCTGGTCTGCTTA  R: GTTTCCTTCTCCGTCGTG |
| IL-6 | F: CTGGCAGAAAACAACCTGAACC  R: TGATTCTCATCAAGCAGGTCTCC |
| IL-10 | F: GCATCCACTTCCCAACCA  R: GCAACAAGTCGCCCATCT |
| TNF-α | F: GCCTCAGCCTCTTCTCCTT  R: GCATTGGCATACCCACTCT |
| IFN-β | F: CGATACCAACAAAGGAGCAG  R: GGTTTCATTCCAGCCAGT |
| CP204L | F: TAATCCGTGTCCCAACTA  R: TCTTACATACCCTTCCACTA |
| β-actin | F: CTCCATCATGAAGTGCGACGT  R: GTGATCTCCTTCTGCATCCTGTC |

F: Forward primer

R: Reverse primer
