# Supplementary material for: African swine fever virus infection enhances CD14-dependent phagocytosis of porcine alveolar macrophages to promote bacterial uptake and apoptotic body-mediated viral transmission
Source: J Virol. 2025 Jun 12;99(7):e00690-25. doi: 10.1128/jvi.00690-25 (PMC12282190; doi:10.1128/jvi.00690-25)
Supplement: Table S2 — siRNA sequences. [file jvi.00690-25-s0010.docx]

**Table S2. siRNA sequences**

| **Gene** | **Sequences 5’ to 3’** |
| --- | --- |
| CD14 | F: GCCUCAAGGUACUGAAAGUTT  R: ACUUUCAGUACCUUGAGGCTT |
| TLR2 | F: GCAGAAAUCACUGACGCAATT  R: UUGCGUCAGUGAUUUCUGCTT |
| CD64 | F: GCAGGAGUAUCUAUCACUATT  R: UAGUGAUAGAUACUCCUGCTT |
| CD48 | F: GACACAAUCUACUUCACUUTT  R: AAGUGAAGUAGAUUGUGUCTT |
| SDC4 | F: GCAGGAACCUGAUGACUUUTT  R: AAAGUCAUCAGGUUCCUGCTT |
| NC | F: UUCUCCGAACGUGUCACGUTT  R: ACGUGACACGUUCGGAGAATT |

F: Forward sequence

R: Reverse sequence
